# Malnutrition risk as a predictor of quality of life and skeletal muscle depletion following upper gastrointestinal cancer diagnosis: A longitudinal analysis

**DOI:** 10.1016/j.jnha.2025.100623

**Published:** 2025-07-01

**Authors:** Lauren Hanna, Kay Nguo, Judi Porter, Daniel Croagh, Catherine E Huggins

**Affiliations:** a Department of Nutrition, Dietetics and Food, Monash University, Clayton, Victoria, Australia; b Department of Nutrition and Dietetics, Monash Health, Monash Medical Centre, Clayton, Victoria, Australia; c Institute for Physical Activity and Nutrition, School of Exercise and Nutrition Sciences, Deakin University, Geelong, Victoria, Australia; d Department of Surgery, School of Clinical Sciences, Monash University, Clayton, Victoria, Australia; e Department of Upper Gastrointestinal Surgery, Monash Health, Clayton, Victoria, Australia; f Global Centre for Preventive Health and Nutrition (GLOBE), Institute for Health Transformation, School of Health and Social Development, Deakin University, Clayton, Victoria, Australia

**Keywords:** Skeletal muscle index, Low muscle mass, Sarcopenia, Health-related quality of life, EORTC QLQ-C30, PG-SGA

## Abstract

**Objectives:**

Low skeletal muscle index (SMI) and radiodensity (SMD) are established prognostic indicators in cancer. This study investigated risk factors for low and decreasing SMI and low SMD in upper gastrointestinal cancer and examined the influence of malnutrition risk on the association between SMI and health-related quality of life (HRQOL).

**Design:**

Longitudinal analysis of randomised controlled trial outcome data.

**Setting:**

Three health services in Victoria, Australia.

**Participants:**

Adults newly diagnosed with oesophageal, gastric or pancreatic cancer.

**Measurements:**

Outcomes assessed at diagnosis, and three- and six-month follow-up. SMI and SMD were assessed via computed tomography imaging analysis, with low values determined using established sex-specific thresholds. Malnutrition risk was assessed using the Patient Generated Subjective Global Assessment (Short Form), and HRQOL with the EORTC QLQ-C30. Multiple logistic regression identified risk factors for low SMI and low SMD at baseline, and SMI decline (≥5%) from baseline to 3 months. Associations between SMI and HRQOL were examined using multiple linear regression, adjusting for malnutrition risk.

**Results:**

Among 105 participants (43% oesophageal, 20% gastric, 37% pancreatic cancer), older age predicted low SMI and low SMD. Low SMI risk was higher in females and lower with higher BMI. At three months, 57% (37/65) experienced ≥5% SMI loss, associated with higher malnutrition risk, higher baseline SMI, and post-diagnosis weight loss. Malnutrition risk was a strong independent predictor of poorer HRQOL score at all timepoints. Lower or decreasing SMI (≥5%) was also independently associated with poorer HRQOL on some scales.

**Conclusion:**

Malnutrition risk independently predicted lower HRQOL and muscle loss, and may confound the relationship between SMI and HRQOL. As a modifiable factor, addressing malnutrition risk could improve HROQL and preserve muscle in upper gastrointestinal cancer.

**Trial registration:**

Australian New Zealand Clinical Trials Registry, 27 January 2017 (12617000152325).

## Highlights

• Female sex increases risk of low SMI, and higher BMI decreases risk of low SMI.
• Older age is a risk factor for low SMI and low SMD.
• Malnutrition risk score at diagnosis predicts subsequent muscle loss.
• Both malnutrition risk score and lower SMI are associated with worse HRQOL.
• Malnutrition risk is a modifiable target for improving HRQOL and maintaining muscle.

## 1 Introduction

Cancers of the upper gastrointestinal tract confer a high disease burden, with gastric, oesophageal and pancreatic cancer among the leading causes of cancer deaths globally [1,2]. Symptoms from both the disease and its treatment cause significant morbidity and impair health-related quality of life (HRQOL) during patients’ often shortened remaining lifespan [3]. As HRQOL is a priority for patients [4], identifying modifiable factors that can be targeted to improve HRQOL is essential.

The adverse impact of low skeletal muscle mass on health outcomes for people with cancer is well established, and it is accepted as a meaningful phenotypic prognostic indicator in many cancers including upper gastrointestinal [5]. Computed tomography (CT) imaging analysis is a gold standard method for skeletal muscle mass assessment in oncology (using skeletal muscle index, SMI) [6]; skeletal muscle radiodensity (SMD) which is distinct from skeletal muscle mass and reduced by fatty infiltration, is also prognostically relevant [7]. Emerging evidence links low skeletal muscle mass with poorer HRQOL in patients with cancer, particular in global and physical functioning domains [8]. However, many studies do not adjust for confounding variables such as nutrition status, limiting the interpretation of this relationship. Poor nutrition status is independently associated with worse HRQOL in cancer [9,10], attributed to factors such as malnutrition-related fatigue and/or nutrition impact symptoms that hinder daily activities [11]. Malnutrition also contributes to skeletal muscle depletion across disease states [12], yet few studies investigating the impact of skeletal muscle stores on health outcomes account for nutrition status. Many of the studies examining CT-derived skeletal muscle stores in cancer are retrospective, limiting the ability to assess malnutrition risk associated with changes in weight and oral intake. Consequently, the role of nutrition as a confounder in the skeletal muscle-HRQOL relationship remains underexplored.

The aetiology of low skeletal muscle mass and skeletal muscle radiodensity is multifactorial, relating to age, lifestyle factors, cancer treatment and systemic inflammation [13,14], however there is a paucity of evidence regarding the influence of patient-reported characteristics on the risks and consequences of low skeletal muscle stores. Few studies have examined risk factors for low SMI or low SMD in upper gastrointestinal cancers [[15], [16], [17], [18]], and nutrition measures have not been included in these analyses. This gap limits the development of effective interventions to prevent or reverse muscle loss and improve HRQOL outcomes. Understanding the role of nutrition status in the associations between skeletal muscle stores and HRQOL outcomes is particularly pertinent in upper gastrointestinal cancers where nutritional decline is common due to tumour- and treatment-related nutrition impact symptoms [19]. Therefore, this study aimed to characterise nutritional and skeletal muscle profiles at diagnosis of upper gastrointestinal cancer and over the six-month post-diagnosis period, in order to: i) identify risk factors for low SMI and SMD at diagnosis, ii) identify risk factors for SMI decrease following diagnosis, and iii) to examine the influence of nutritional condition on the association between skeletal muscle stores and HRQOL.

## 2 Methods

### 2.1 Study design and participant selection

This study is a secondary analysis of prospectively collected data from a three-arm single-blinded randomised controlled trial (RCT) which investigated the effect of early and intensive nutrition intervention on quality adjusted life years lived, in participants with newly diagnosed upper gastrointestinal cancers [19,20]. This RCT was registered with the Australian New Zealand Clinical Trials Registry (ANZCTR) on 27th January 2017 (12617000152325) and was approved by the Monash Health Human Research Ethics committee (RES-16-0000434A). Patients aged ≥18 years were eligible for participation in the RCT within four weeks of a histologically confirmed diagnosis of a new primary cancer of the oesophagus, stomach or pancreas (any stage), for which any active treatment was planned (curative or palliative intent). Individuals were excluded if chemo- and/or radiotherapy treatment had already commenced (although those who had undergone urgent surgical resection of their tumour were eligible), if there was insufficient cognition or knowledge of the English language to provide informed consent, or if they were receiving end of life care. Participants were recruited from three health services in Victoria, Australia between April 2017 and July 2019. Participants (n = 111) were randomised to receive an early, intensive nutrition counselling intervention delivered via 1) telephone or 2) mobile application (‘mobile app’), or 3) to receive usual nutrition care (control group) for an 18-week period which commenced at diagnosis of upper gastrointestinal cancer, prior to any chemo- or radiotherapy treatment. Outcomes were assessed at baseline and 3-, 6- and 12-months. Demographic information was collected through retrospective medical record review. Cancer stage was classified according to the 2017 National Comprehensive Cancer Network (NCCN) guidelines [21].

### 2.2 Skeletal muscle assessment by computed tomography

Contrast-enhanced diagnostic CT imaging was undertaken as close as possible to baseline and 3-, 6- and 12-month follow-up time points. If routine diagnostic CT imaging was not available at baseline, then participants were excluded from this secondary analysis. Skeletal muscle assessment was conducted by a certified researcher using SliceOmatic 5.0 Rev-7 software (Tomovision, Canada), using transverse cross-sectional images at the level of the third lumbar vertebra. To distinguish skeletal muscle, the range of -29 to 150 Hounsfield Units (HU) was used [22]. The skeletal muscle index (SMI) was determined by dividing cross-sectional skeletal muscle area by participant height (cm^2^/m^2^). Participants were classified as having low or normal SMI using the Martin et al. 2013 cut point [23]. For participants where CT imaging was available at a follow-up time point (3-, 6- or 12-months) in addition to baseline, change in SMI from baseline was calculated. The impact of SMI decrease (categorised as ≥2%, ≥5%, ≥10% and ≥20%) on HRQOL was analysed at three months. Skeletal muscle radiodensity (SMD) was measured as the mean radiodensity in Hounsfield Units (HU) of the cross-sectional skeletal muscle area, with participants grouped by low or normal SMD using the Martin et al. cut point [23].

### 2.3 Nutrition status

Body weight was assessed using documented weight in medical records where available. If this was not available, self-reported weight measured by participants in their homes was used. The Patient-Generated Subjective Global Assessment Short Form (PG-SGA_SF_), version 3.22.15 (English) was used to assess malnutrition risk at all study time points [24] and was analysed as both a continuous and categorical variable. For the categorical analysis of PG-SGA_SF_ scores, participants were grouped according to the recommended nutritional triage system of the PG-SGA tool (score 0−1 (no risk) vs. score ≥2, ≥4, ≥9) [24], and through dichotomisation of PG-SGA_SF_ scores (<9 or ≥ 9), a threshold associated with cachexia, unfavourable hospital length of stay, and poor survival outcomes [24,25]. Presence of cachexia was defined according the 2011 consensus definition by Fearon et al. [26]: weight loss >5% over six months, a BMI <20 kg/m^2^ along with weight loss >2%, or SMI of <55 cm^2^/m^2^ in males or <39cm^2^/m^2^ in females, along with weight loss >2%.

### 2.4 Health-related quality of life assessment

HRQOL was assessed at all study timepoints using the European Organisation for Research and Treatment of Cancer Quality of Life Questionnaire – Core 30 (EORTC QLQ-C30) [27]. Scores for the global scale, and the five functional scales were included in this analysis, as they capture the multiple dimensions of HRQOL [27]. The three symptom scales, and six symptom single items of the EORTC QLQ-C30 were not analysed individually. Instead, the summary score of the EORTC QLQ-C30 established in 2016, which incorporates all items of the original tool except the global scale and financial difficulties item [28], was included in the analysis to explore the association of this summary measure of HRQOL with skeletal muscle stores.

### 2.5 Statistical analysis

Data were tested for normality using the Shapiro-Wilk test. Spearman’s rank correlation or Pearson’s correlation test were used to determine the association between continuous variables. The chi-square test was used to analyse frequency differences between groups of participants. To compare distribution of continuous variables between groups the independent samples t-test, one-way analysis of variance Mann-Whitney U test or Kruskal–Wallis test was used depending on normality of data and number of groups. The Jonckheere-Terpstra test for ordered alternatives was used to examine the trend of median SMI across nutritional triage groups with increasing malnutrition risk.

Logistic regression was used to determine odds ratios and 95% CIs for variables considered to be associated with low SMI, low SMD, and SMI decrease of ≥5% from baseline to three months (a threshold chosen due to reported association with survival [29]); variables with a statistical significance of *p* < 0.25 on univariate analysis were included in multiple logistic regression models [30]. Linear regression was used to examine the association between skeletal muscle stores and continuous measures of HRQOL taken at each time point; variables with a statistical significance of *p* < 0.05 were included in standard multiple linear regression models. For all multivariate linear regression analyses, age, sex, cancer type (pancreatic vs oesophageal and gastric) and PG-SGA_SF_ covariates were selected *a priori*, as described in the published RCT protocol [19], and skeletal muscle variables were included to address the aims of this study. All variables included in multivariate regression analyses were checked for multicollinearity before analysis. The nutrition intervention delivered as part of the RCT did not have a statistically significant impact on HRQOL [20] or on skeletal muscle loss (data not shown), therefore it was not included as a cofactor in the analyses. Statistical analyses were conducted using SPSS software (Version 27, IBM, Armonk, USA). Listwise deletion was used to handle missing data. All statistical tests were two-sided, with a *p* value of < 0.05 indicating statistical significance.

## 3 Results

### 3.1 Participant selection

**Fig. 1:**
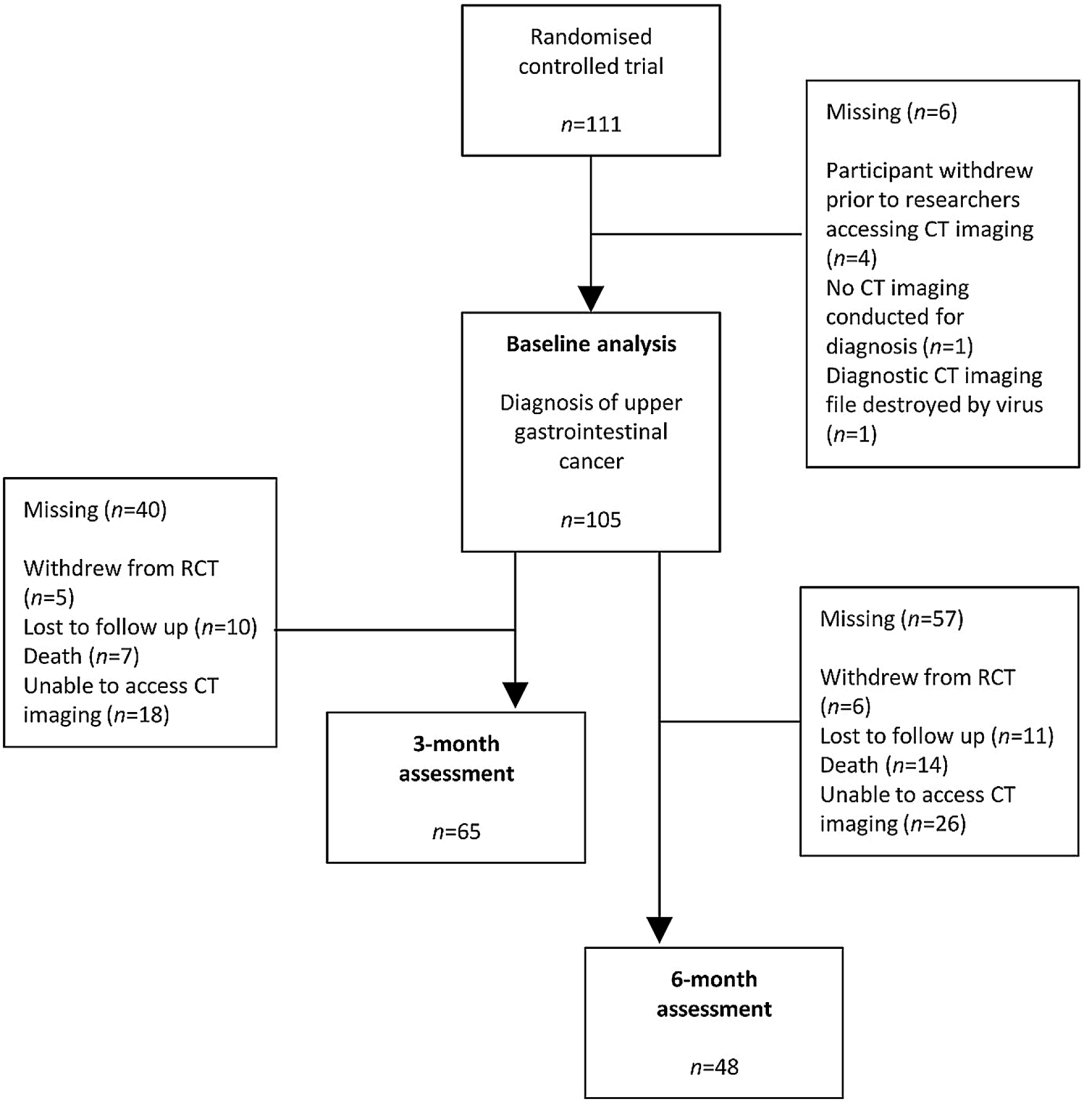
Flow chart of sample selection for the present analysis (n = 105), derived from the cohort of participants enrolled in the randomised controlled trial (n = 111) [19].

A flowchart for the recruitment, selection and analyses of participants (*n* = 105), is presented in Fig. 1. Diagnostic CT images were available for 105 participants (95% of RCT cohort), which had been conducted a median of 12 days (IQR 5–23) prior to HRQOL assessment. Reasons for missing CT imaging (*n* = 6) are outlined in Fig. 1. There were no significant differences in age, gender, cancer type, stage of disease, BMI, or PG-SGA_SF_ score between those included in this study (*n* = 105), and those excluded due to lack of CT imaging (*n* = 6) (Appendix A). Excluded participants had a lower median cognitive function scale HRQOL score compared to the included participants (50 (29.2–87.5) vs. 83.3 (66.7−100), *p* = 0.023); there were no other differences in HRQOL between included and excluded participants. Of the 105 included participants, CT imaging and HRQOL data were available for 65 participants at three months, and for 48 participants at the six-month time point. Due to the variation in availability of data, the participant sample at each time point represents a distinct sub-cohort of the baseline cohort. Data were available for only 32 participants at 12 months; therefore, this time point was not included in the present study.

### 3.2 Treatment status at enrolment

Treatment plans at diagnosis included chemotherapy for 100 participants (95.2%) and radiotherapy for 49 participants (46.7%). No participants had commenced chemo- or radiotherapy prior to the CT imaging used for baseline skeletal muscle analysis, or completion of the baseline malnutrition risk or HRQOL assessment. Surgical resection was planned for 57 participants (54.3%); for eight participants (7.6%), surgery was pending response to neo-adjuvant chemotherapy. Four participants had undergone surgical resection of their tumour prior to completion of baseline malnutrition risk and HRQOL assessments, and for one of these participants this surgery also preceded the CT imaging used for body composition analysis by nine days. As surgery is known to impact on skeletal muscle stores [15], a subgroup analysis with removal of the participant with surgical resection prior to CT imaging (*n* = 1) was undertaken (data not shown); this did not change the significance of any of the findings of this study, therefore the data presented includes this participant.

### 3.3 Participant characteristics

Demographic, phenotypic, and malnutrition risk data are presented in Table 1. Over two-thirds of participants were male, with oesophageal cancer the most frequently occurring cancer type. Approximately half of participants had resectable or borderline resectable disease, 21% had metastatic disease at diagnosis*.* Seventy percent of participants were cachectic at diagnosis, and just under half had a PG-SGA_SF_ score of ≥ 9 indicating a critical need for nutrition intervention [24].

**Table 1:** Demographic, nutritional status and health-related quality of life of participants at diagnosis of upper gastrointestinal cancer (baseline), and at 3- and 6-months post diagnosis.

	Baseline n = 105	3 months n = 65	6 months n = 48
***Demographics***			
Age (years)^a^	65.9 (10.0)	66.4 (10.0)	64.6 (11.0)
Sex, *n* (%)			
Male	71 (67.6)	44 (67.7)	35 (72.9)
Female	34 (32.4)	21 (32.3)	13 (27.1)
Cancer type, *n* (%)			
Oesophageal	45 (42.9)	30 (46.2)	25 (52.1)
Gastric	21 (20.0)	13 (20.0)	6 (12.5)
Pancreatic	39 (37.1)	22 (33.8)	17 (35.4)
Clinical stage, *n* (%)			
Resectable	46 (43.8)	29 (44.6)	19 (39.6)
Borderline resectable	6 (5.7)	6 (9.2)	2 (4.2)
Locally advanced	31 (29.5)	18 (27.7)	15 (31.3)
Metastatic	22 (21.0)	12 (18.5)	12 (25.0)
BMI (kg/m^2^)	25.1 (22.4−28.0)	24.8 (22.9−28.2)	24.4 (3.5)^a^
***Weight loss in 6 months to diagnosis***			
Weight loss category, *n* (%)			
Gain or <2% loss	28 (26.7)	25 (38.5)	17 (35.4)
2−4.9% loss	13 (12.4)	15 (23.1)	11 (22.9)
5−9.9% loss	28 (26.7)	18 (27.7)	9 (18.8)
10−19.9% loss	27 (25.7)	7 (10.8)	9 (18.8)
≥20% loss	9 (8.6)	–	2 (4.2)
***Malnutrition screening***			
PG-SGA_SF_ score	8 (2−14)	8.5 (2−12)^b^	4 (2.3−9)
PG-SGA_SF_ category, *n* (%)			
0−1	20 (19.0)	12 (18.5)^b^	7 (14.6)
2−3	14 (13.3)	8 (12.3)^b^	16 (33.3)
4−8	20 (19.0)	12 (18.5)^b^	11 (22.9)
≥9	51 (48.6)	32 (50.0)^b^	14 (29.2)
***Skeletal muscle stores***			
SMI (cm^2^/m^2^)	44.5 (37.6−52.1)	44.1 (8.8)^a^	44.5 (8.4)^a^
SMI change from baseline (%)	–	−5.6 (7.6)^a^	−4.8 (10.4)^a^
SMI change from baseline, n (%)			
Gain or <0% decrease	–	14 (21.5)	17 (35.4)
0−4.9% decrease	–	14 (21.5)	8 (16.7)
5−9.9% decrease	–	21 (32.3)	9 (18.8)
10−19.9% decrease	–	14 (21.5)	10 (20.8)
≥20% decrease	–	2 (3.1)	4 (8.3)
SMD (mean HU)^a^	35.3 (12.7)	32.2 (8.6)^a^, ^c^	35.6 (9.9)^a^, ^d^
***Presence of cachexia, n (%)***	74 (70.5)	–	27 (56.3)
***Health-related quality of life***			
EORTC QLQ-C30 score			
Global	66.7 (41.7−83.3)	66.7 (41.7−75)^e^	66.7 (58.3−81.3)
Physical	86.7 (66.7−100)	80 (60−93.3)	80 (61.7−93.3)
Role	83.3 (33.3−100)	66.7 (33.3−83.3)	66.7 (50−100)
Cognitive	83.3 (66.7−100)	83.3 (66.7−100)^e^	83.3 (66.7−100)
Social	83.3 (50−100)	83.3 (50−100)^e^	83.3 (66.7−100)
Emotional	75 (58.3−91.7)	83.3 (66.7−91.7)^e^	83.3 (66.7−91.7)
Summary	81.1 (66.0−91.5)	74.8 (61.5−87.5)^a^, ^e^	80.9 (68.9−86.8)^a^

BMI body mass index; PG-SGA_SF_ Patient Generated Subjective Global Assessment Short Form; SMD skeletal muscle radiodensity; SMI skeletal muscle index; all continuous data presented as median (interquartile range) unless otherwise indicated.

a Mean (standard deviation).

b n = 64, missing data for one participant due to not completing PG-SGA_SF_ form.

c n = 63, missing data for two participants due to contrast allergies.

d n = 45, missing data for three participants due to contrast allergies.

e n = 63, missing data for two participants due to not completing back of form.

### 3.4 Risk factors for low skeletal muscle stores at diagnosis of upper gastrointestinal cancer

#### 3.4.1 Skeletal muscle index

The association between baseline demographic, phenotypic and malnutrition risk variables with risk of low SMI at diagnosis of upper gastrointestinal cancer is presented in Table 2. Fifty-nine participants (56%) had low SMI at diagnosis. More females had low SMI at diagnosis than males (79% vs 45%, p < 0.05), with female sex independently associated with over four-times greater odds of having low SMI at diagnosis (Table 2). Age was weakly, negatively correlated with SMI (*r* = −0.262, *p* = 0.007); participants with low SMI were older than those with normal SMI, with a small increase in odds of low SMI observed for every additional year. Cancer type and cancer stage variables were not associated with risk of low SMI in multivariate models adjusting for age, sex, cancer type and BMI (Table 2).

**Table 2:** Association between baseline demographic, phenotypic and malnutrition risk variables with risk of low SMI at diagnosis of upper gastrointestinal cancer.

	Low SMI n = 59	Normal SMI n = 46	p	Univariate OR (95% CI)	*p*	Multivariate OR (95% CI)	***p***
Age (years)	68.0 (10.0)^a^	63.1 (9.2)^a^	0.011*	1.05 (1.01−1.10)	0.013*	1.07 (1.02−1.13)	0.007 d, *
Sex, n (%)							
Male	32 (54.2)	39 (84.8)	0.002^b^, *	1.0 (ref)		1.0 (ref)	
Female	27 (45.8)	7 (15.2)		4.70 (1.81−12.20)	0.001*	4.73 (1.59−14.08)	0.005^d^, *
Cancer type, n (%)							
Oesophageal/gastric	33 (55.9)	33 (71.7)	0.144	1.0 (ref)		1.0 (ref)	
Pancreatic	26 (44.1)	13 (28.3)		2.00 (0.88−4.55)	0.098	0.82 (0.27−2.51)	0.731^d^
Cancer stage, n (%)							
I-II (resectable/borderline)	23 (39.0)	29 (63.0)	0.024^c^, *	1.0 (ref)		1.0 (ref)	
III-IV (unresectable)	36 (61.0)	17 (37.0)		2.67 (1.21−5.91)	0.015*	2.01 (0.79−5.14)	0.144^d^
BMI (kg/m^2^)	23.8 (21.3−26.8)	26.6 (24.2−30.0)	<0.001*	0.82 (0.74−0.92)	<0.001*	0.85 (0.75−0.96)	0.008^d^*
Weight loss during 6 months pre-diagnosis (%)	6.9 (2.6−12.5)	6.3 (0−10.4)	0.203	1.05 (0.99−1.11)	0.131	1.05 (0.96−1.14)	0.278^d^
PG-SGA_SF_ score, n (%)	9.0 (3.0−15.0)	5.5 (1.0−11.5)	0.048*	1.07 (1.00−1.14)	0.039*	1.06 (0.97−1.16)	0.228^e^
< 9	27 (45.8)	27 (58.7)	0.263	1.0 (ref)		1.0 (ref)	
≥ 9	32 (54.2)	19 (41.3)		1.68 (0.77−3.67)	0.190	1.24 (0.45−3.41)	0.695^e^

n = 105; continuous data reported as median (interquartile range) unless otherwise indicated; BMI body mass index; PG-SGA_SF_ Patient Generated Subjective Global Assessment Short Form.

a mean (standard deviation).

b significant difference between groups for both males and females (p < 0.05).

c significant difference between groups for both Stage I-II and Stage III and IV (p < 0.05).

d model includes age, female sex, cancer type, cancer stage, BMI, and prior weight loss (variables presented in this table).

e model adjusts for age, female sex, cancer type, cancer stage, and BMI (variables not presented);

* Indicates statistically significant result, p < 0.05.

BMI was positively correlated with SMI (*r* = 0.678, *p* < 0.001), and a higher BMI was independently associated with lower odds of having low SMI (Table 2). There was a negative correlation between SMI and continuous nutrition-related measures: percentage weight loss in previous six months (*r* = −0.237, *p* = 0.015), and PG-SGA_SF_ score (higher score indicates higher risk) (*r* = −0.268, *p* = 0.006). When participants were grouped by PG-SGA_SF_ score according to the tool’s recommended triage categories, a Jonckheere-Terpstra test for ordered alternatives showed a statistically significant trend of a lower SMI as PG-SGA_SF_ score increased, *J* = 1488, *z* = −2.20, *p* = 0.028, *r* = −0.21) (Appendix C). In multivariate analyses however, neither high malnutrition risk score (PG-SGA_SF_ ≥9) nor weight loss in the six months prior to diagnosis were independent risk factors for low SMI (Table 2).

#### 3.4.2 Skeletal muscle radiodensity

The analyses for the association between baseline demographic, phenotypic and malnutrition risk variables with risk of low SMD at diagnosis of upper gastrointestinal cancer are presented in Appendix D. Fifty-eight participants (55%) had low SMD at diagnosis. There was no association between sex and SMD. Age was strongly, negatively correlated with SMD (*r* = −0.517, *p* < 0.001); participants with low SMD were older than those with normal SMD (70.6 ± 7.9 vs. 60.0 ± 9.0 years, p < 0.001), with a small increase in risk of low SMD observed for every additional year (OR 1.16 (95% CI 1.09–1.24), p < 0.001). Cancer type and cancer stage variables were not associated with risk of low SMD in multivariate models adjusting for age, sex, cancer type and BMI.

There was a weak negative correlation between BMI and SMD (*r* = −0.207, *p* = 0.034), however BMI was not associated with odds of having low SMD (OR 0.99 (0.91–1.07), p = 0.759). There was a small positive correlation between SMD and percentage weight loss in previous six months (*r* = 0.284, *p* = 0.003), but not with PG-SGA_SF_ score (*r* = 0.116, *p* = 0.237); neither of these variables were associated with risk of low SMD in multivariate analyses.

### 3.5 Risk factors for skeletal muscle loss over three months from diagnosis

Thirty-seven participants (57%) lost greater than five percent of SMI from diagnosis (Table 3). In univariate analysis, these participants had a higher baseline SMI than those who lost less than five percent SMI, but no other significant demographic or clinical differences. In the multivariate analysis, higher SMI at diagnosis, percentage weight loss from diagnosis, and high malnutrition risk at baseline (PG-SGA_SF_ score ≥9) were independent risk factors for losing over five percent SMI in the three months following upper gastrointestinal cancer diagnosis, adjusting for sex.

**Table 3:** Association between baseline demographic, phenotypic and malnutrition risk variables with risk of SMI decrease ≥5% over three months following diagnosis of upper gastrointestinal cancer.

	SMI decrease ≥5% n = 37	SMI decrease <5% n = 28	*p*	Univariate OR (95% CI)	*p*	Multivariate OR (95% CI)	*p*
Age at baseline (years)^a^	65.6 (8.8)	66.8 (11.4)	0.632	0.988 (0.939−1.038)	0.627		
Sex, n (%)							
Male	29 (78.4)	15 (53.6)	0.064	1.0 (ref)		1.0 (ref)	
Female	8 (21.6)	13 (46.4)		0.318 (0.108−0.936)	0.038*	0.709 (0.167−3.015)	0.642
Cancer type, n (%)							
Oesophageal/gastric	24 (64.9)	19 (67.9)	1.000	1.0 (ref)			
Pancreatic	13 (35.1)	9 (32.1)		1.114 (0.404−3.240)	0.801		
Cancer stage, n (%)							
I-II (resectable/borderline)	20 (54.1)	15 (53.6)	1.000	1.0 (ref)			
III-IV (unresectable)	17 (45.9)	13 (46.4)		0.981 (0.366−2.626)	0.969		
Baseline SMI (cm^2^/m^2^)	49.4 (42.0−56.3)	42.4 (35.3−46.9)	0.002*	1.095 (1.029−1.166)	0.004*	1.117 (1.029−1.212)	0.008*
Weight loss from baseline (%)^b^	4.3 (1.3−9.1)	2.3 (0−6.3)	0.059	1.147 (1.003−1.312)	0.045*	1.178 (1.006−1.379)	0.042*
Baseline PG-SGA_SF_ score	9 (2.5−13.5)	3 (1−13)	0.239	1.039 (0.962−1.122)	0.329		
< 9	18 (48.6)	19 (67.9)	0.195	1.0 (ref)		1.0 (ref)	
≥ 9	19 (51.4)	9 (32.1)		2.228 (0.802−6.193)	0.124	3.933 (1.125−13.748)	0.032*

n = 65; *continuous data reported as median (interquartile range) unless otherwise indicated;* PG-SGA_SF_ Patient Generated Subjective Global Assessment Short Form; SMI skeletal muscle index.

a mean (standard deviation).

b weight gain analysed as 0% weight loss.

* indicates statistically significant result, p < 0.05.

### 3.6 Relationship between skeletal muscle stores and health-related quality of life

#### 3.6.1 Baseline

Univariate relationships between skeletal muscle stores (analysed as both a continuous and a categorical variable) and HRQOL at diagnosis of upper gastrointestinal cancer are presented in Table 4. Higher SMI was associated with higher (better) HRQOL scores for the physical function, role function and summary scales; when dichotomised to low or normal SMI, HRQOL was significantly lower in participants with low SMI for the physical function scale only. There was no correlation between HRQOL and SMD (muscle quality), and no difference in HRQOL between participants with low or normal SMD.

**Table 4:** Univariate relationship between EORTC QLQ-C30 global and functioning scale scores, with skeletal muscle stores analysed as a continuous and a categorical variable, at diagnosis of upper gastrointestinal cancer.

	Skeletal muscle index (SMI)					Skeletal muscle radiodensity (SMD)				
	*r*	*p*	Low SMI (n = 59)	Normal SMI (n = 46)	*p*	*r*	*p*	Low SMD (n = 58)	Normal SMD (n = 47)	*p*
Global	0.112	0.255	58.3 (33.3−83.3)	66.7 (50−83.3)	0.231	−0.068	0.492	66.7 (33.3−83.3)	62.5 (50−83.3)	0.910
Physical	0.250	0.010*	80.0 (60−93.3)	93.3 (73.3−100)	0.033*	0.054	0.582	86.7 (66.7−93.3)	93.3 (73.3−100)	0.225
Role	0.225	0.021*	66.7 (33.3−100)	83.3 (50−100)	0.055	−0.073	0.460	75.0 (33.3−100)	83.3 (33.3−100)	0.687
Cognitive	−0.046	0.638	83.3 (66.7−100)	83.3 (66.7−100)	0.782	0.003	0.974	83.3 (66.7−100)	83.3 (66.7−100)	0.994
Social	0.024	0.811	83.3 (50−100)	83.3 (66.7−100)	0.968	−0.174	0.075	83.3 (62.5−100)	83.3 (50−100)	0.253
Emotional	0.044	0.655	75.0 (50−91.7)	83.3 (64.6−91.7)	0.489	−0.126	0.199	83.3 (66.7−91.7)	66.7 (50−83.3)	0.145
Summary	0.218	0.025*	35.7 (33.6−36.9)	36.0 (35.0−37.2)	0.119	−0.041	0.675	35.6 (33.9−36.8)	36.2 (34.6−37.5)	0.116

n = 105; *r* correlation coefficient; *p* p-value; EORTC QLQ-C30 European Organisation for Research and Treatment of Cancer Quality of Life Questionnaire – Core 30

* Indicates statistically significant result, p < 0.05.

The contribution of SMI to variation in HRQOL scores at diagnosis, adjusted for age, sex, cancer type and PG-SGA_SF_ score are presented in Appendix B (Tables B1 to B7). SMI as a continuous variable was negatively associated with cognitive function (β −0.370 (95% CI −1.166, −0.248), p = 0.003, Table B5); no independent associations were observed for the other HRQOL scales. There was no independent association between low SMI and any HRQOL score at diagnosis of upper gastrointestinal cancer in multivariate analysis. Malnutrition risk assessed using the PG-SGA_SF_ tool was an independent predictor of lower HRQOL in most multivariate models, regardless of whether the threshold used was a score of ≥2, 4, or 9; effect sizes increased with higher PG-SGA_SF_ score thresholds (i.e. for PG-SGA_SF_ score ≥2, summary HRQOL score β-0.308, 95%CI −22.252, −5.570, p = 0.001, and for PG-SGA_SF_ score ≥9, summary HRQOL score β-0.524, 95%CI −24.342, −12.848, p < 0.001 (Table B7). SMD was not included in these analyses due to lack of association with HRQOL demonstrated in Table 4.

#### 3.6.2 Three-months and six-months post diagnosis

The association of SMI to HRQOL at three- and six-month time points are presented in Appendix B. At three months (n = 65), there was no univariate association between SMI (continuous) and any HRQOL scale. At six months (n = 48), SMI (continuous) was independently associated with physical function (β 0.373 (95% CI 0.172, 1.616), p = 0.017, Table B16) and social function (β 0.360 (95% CI −0.086, 2.009), p = 0.033, Table B18), but no association was demonstrated with the other HRQOL scales. In these models, malnutrition risk using one or more PG-SGA_SF_ thresholds (≥2, 4, or 9) was an independent predictor of lower HRQOL in all scales except emotional function at three months.

Low SMI was not associated with any HRQOL scale at three months; at six months, low SMI was independently associated with the summary HRQOL score (β −0.250 (95% CI −13.335, −0.149), p = 0.045, Table B21), but no association was demonstrated with the other HRQOL scales. In these models, malnutrition risk using one or more PG-SGA_SF_ thresholds (≥2, ≥4, or ≥9) was an independent predictor of lower HRQOL in all scales except emotional function at three months.

The association between SMI decrease (≥2%, 5%, 10% and 20%) from baseline to three months was also examined in regression models adjusted for age, sex, cancer type, baseline PG-SGA_SF_ score and baseline SMI. An SMI decrease from diagnosis of ≥2% was independently associated with lower role function (β −0.314 (95% CI −42.610, −4.186), p = 0.018), social function (β −0.396, (95%CI −50.800, −10.630), p = 0.003), and summary HRQOL (β −0.275 (95% CI −20.988, −0.773), p = 0.035). An SMI decrease of ≥5% was associated with lower global HRQOL (β −0.311 (95% CI −26.765, −3.680), p = 0.011), as well as role function, social function, and summary HRQOL. An SMI decrease of ≥10% was associated with lower cognitive function (β −0.300 (95% CI −29.962, −2.660), p = 0.020), as well as global HRQOL, role function, social function, and summary HRQOL. Two participants (3.1%) experienced a decrease in SMI of ≥20%; this was not associated with HRQOL in any scale. In these models adjusting for SMI decrease from baseline to three months, malnutrition risk using one or more PG-SGA_SF_ thresholds (≥2, ≥4, or ≥9) was an independent predictor of lower HRQOL in all scales except emotional function and cognitive function.

## 4 Discussion

This study examined risk factors for low SMI and low SMD at diagnosis of upper gastrointestinal cancer, SMI decrease post-diagnosis, and associations between skeletal muscle stores and HRQOL (adjusted for malnutrition risk) over six months from diagnosis. Independent predictors of low SMI at diagnosis were female sex, older age, and lower BMI, while only older age predicted low SMD risk. Risk factors for SMI decrease ≥ 5% in the three months post-diagnosis were high malnutrition risk or higher SMI at diagnosis, and percentage weight loss during this period. SMI was negatively associated with cognitive function at baseline, and positively associated with physical function, social function and summary HRQOL at six months. A decrease in SMI as small as 2% from baseline to three months negatively impacted HRQOL at three months in multiple scales. Malnutrition risk assessed using the PG-SGA_SF_ was a strong independent predictor of lower HRQOL at every time point.

Low SMI and low SMD were prevalent at upper gastrointestinal cancer diagnosis, affecting 56% and 55% respectively. The identification of female sex as a risk factor for low SMI and older age as a risk factor both low SMI and SMD support findings in colorectal [31] and pancreatic cancer [32,33]; to our knowledge, this is the first study to investigate risk factors in both early and advanced oesophageal or gastric cancers. Higher BMI was protective against low SMI, consistent with current evidence [17,[33], [34], [35]]. An inflammatory response related to both ageing and immune system mobilisation may play a role in these findings, as systemic inflammation impairs muscle synthesis and promotes catabolism (reducing SMI) as well as facilitating fatty infiltration of muscle (reducing SMD) [13,14]. Although inflammation data was not available for incorporation into the present study, the identification of other demographic parameters increasing risk of poor outcomes related to low SMI and/or SMD can be used to inform screening program development.

While malnutrition risk score and weight loss prior to diagnosis correlated with SMI in univariate analyses, they were not independently associated with low SMI in the multivariate models. The PG-SGA_SF_ tool captures dietary adequacy and presence (and number) of symptoms impacting oral intake and functional capacity in addition to weight loss; at diagnosis of upper gastrointestinal cancer these factors may have been present long enough to increase malnutrition risk, but not long enough to cause a discernible reduction in SMI below the ‘low SMI’ threshold. In contrast to our findings, Kubrak et al. [36] identified weight loss grade (incorporating BMI) as an independent predictor of low SMI in patients with head and neck cancers (n = 1231). This discrepancy may relate to the larger sample size and BMI inclusion in the Kubrak et al. study; BMI was also found in our study to be related to risk of low SMI. Souza et al. [34] identified malnutrition presence and severity (using the full PG-SGA tool) as a risk factor for sarcopenia defined as low SMI plus low muscle strength or function, in a study of patients with colorectal cancer (n = 195); the use of related but divergent measures in that study may explain the difference in result.

At diagnosis, low SMI was evident in some participants with low or no malnutrition risk (Table 2), consistent with other studies in various cancer types [34,37,38]. This underscores the multifactorial nature of skeletal muscle depletion which may precede diagnosis or the onset of nutrition impact symptoms. Whilst malnutrition risk and low SMI often overlap, this study reinforces that malnutrition screening tools alone cannot reliably detect all cases of low skeletal muscle mass, and both should be assessed separately. Moreover, low SMD confers its own morbidity and mortality risks [7] and can only be detected with radiological imaging, highlighting the value of combining CT imaging analysis with nutrition screening for comprehensive risk assessment in upper gastrointestinal cancer.

SMI decrease post-diagnosis was common in this cohort: 57% of participants lost ≥ 5% SMI within three months, a condition associated with significantly shorter survival in colorectal cancer (HR 2.67 (95% CI 1.59–4.47), p = 0.002, n = 226) [29]. Higher SMI or PG-SGA_SF_ score ≥ 9 at diagnosis, and post-diagnosis weight loss independently predicted risk of SMI loss ≥ 5% at three months. Similar findings have been reported in colorectal cancer (n = 300) [39] and non-small cell lung cancer (where higher BMI was also a risk factor, n = 64) [40]. This highlights that a ‘normal’ or ‘high’ SMI at diagnosis should not be ignored as the risk of muscle loss is greater for these patients [39], and also underscores the importance of ongoing monitoring.

A high PG-SGA_SF_ score at diagnosis, while not indicative of low SMI, was linked to a nearly four-fold increase in risk of SMI loss ≥5% in the following three months. This novel finding enhances our understanding of post-cancer diagnosis skeletal muscle depletion risk. Effective interventions to reduce malnutrition risk and weight loss may help to preserve skeletal muscle, although the impact of systemic inflammation and potential refractory cachexia must also be considered [41]. Further research is required to define malnutrition risk thresholds for skeletal muscle depletion, and determine whether interventions that effectively reduce malnutrition risk are capable of preserving skeletal muscle stores in people with varying severity of cancer cachexia both with and without inflammation [26].

The association observed at baseline between higher SMI and lower cognitive function HRQOL was unexpected. Prior studies have linked lower skeletal muscle mass or sarcopenia with worse cognitive function [42,43], contrasting with our results. Cognitive decline following a cancer diagnosis is not uncommon, possibly related to oxidative stress, inflammatory pathways or vascular changes [44]. It is possible that those with higher SMI at diagnosis had better pre-diagnosis functional capacity and shorter duration of illness; in these individuals the distress of diagnosis or undergoing a more aggressive treatment regimen may have had a greater impact on cognitive function. A similar unexpected finding was reported in a cohort of 51 older healthy males, where a smaller neck muscle cross-sectional area assessed through magnetic resonance imaging analysis was associated with higher cognition; this was hypothesised to relate to level of manual work undertaken by participants, but lack of occupational data meant this theory could not be verified [45].

The negative association between SMI and physical function at six months aligns with our previous meta-analysis demonstrating worse physical function HRQOL for those with low SMI (MD −0.4, 95% CI −0.74, −0.05, p = 0.02, n = 225) [8]. The previous review identified that a lack of statistical adjustment for confounding factors by individual studies limited the interpretation of an association between CT-derived low SMI and lower HRQOL scores which has been addressed in the present study. The underlying mechanism for the association between skeletal muscle mass and HRQOL is often attributed to impaired strength and function [15,16]. A challenge in the interpretation of this relationship is the unequal timeframe (and therefore aetiology) for decline in these measures, which cannot be fully accounted for in cross-sectional studies [46]. HRQOL assessed using the EORTC QLQ-C30 captures participants’ observation of their life and experiences over the limited period of the past week, whereas skeletal muscle depletion can occur slowly or rapidly, over weeks, months or years, affected by factors such as activity level and nutrition status, both premorbid or due to the presence of malignancy [47]. Our results show that SMI loss within three months post-diagnosis independently predicted of lower HRQOL across several scales, even after adjusting for baseline malnutrition risk and other demographic variables. Similar findings have been reported in unadjusted analyses, where SMI decrease was linked to lower HRQOL in gastric and oropharyngeal cancers [15,48], and skeletal muscle maintenance has been linked to better HRQOL scores compared with skeletal muscle loss in colorectal cancer [42]. It is possible therefore that low SMI at cancer diagnosis with its unknown origination and duration does not directly influence HRQOL, but that when skeletal muscle loss is confirmed and quantified in longitudinal studies, the link to poor HRQOL is clearer. Systemic inflammation may influence this relationship [49], and future studies should incorporate inflammation as a variable in statistical analyses.

Higher malnutrition risk was a strong independent contributor to lower HRQOL at diagnosis of upper gastrointestinal cancer and in the following six months, consistent with existing evidence in cancer [9,10]. Studies have shown symptoms such as swallowing problems and mouth sores impair HRQOL in patients with head and neck cancer [50], and cancer treatment side effects such as chemotherapy-induced nausea are associated with a clinically meaningful decrease in HRQOL in patients with mixed cancer types [51]. Evidence suggests that as the number of nutrition impact symptoms increases, risk of malnutrition increases [52] and HRQOL decreases [53]. The adequacy of treatment of nutrition impact symptoms is understudied, however available evidence indicates that many nutrition impact symptoms are not sufficiently controlled with appropriate prescription of medication [54], and that chemotherapy induced nausea and vomiting is underestimated (and therefore undertreated) by clinicians [55]. Effective management of nutrition impact symptoms is therefore critical for optimising HRQOL during cancer treatment.

Strengths of this study include the use of validated tools and techniques to measure skeletal muscle mass and radiodensity, cancer-specific HRQOL, and malnutrition risk, all conducted by a single researcher. Additionally, there was greater homogeneity in cancer treatment status of participants in this study at baseline, compared with previous studies [8]: all participants were naïve to chemo- or radiotherapy at the time of both CT imaging and HRQOL assessment. A limitation of this study was the need to retrospectively obtain CT imaging for analysis of skeletal muscle stores, resulting in some missing data; non-clinical CT scans are not undertaken for research as they expose participants to radiation. The exclusion of six participants due to a lack of available CT imaging was unavoidable but raises the potential for selection bias. As a telehealth study, the need to rely on either medical record documentation or participants’ home scales for measures of body weight limits the precision of measurements reported due to potential variance between scales. An additional limitation was the amount of missing data which increased with each follow-up time point due to participant death, withdrawal from the primary study, inability to contact participants, or non-existence of/lack of access to CT imaging at a time that corresponded to follow up HRQOL and nutrition assessment. It was beyond the scope of this study to control for factors such as participant comorbidities including systemic inflammation, physical activity level, surgical intervention or chemotherapy regime, dose or duration; these factors have individual associations to skeletal muscle, HRQOL, and/or malnutrition risk, and are additional potential confounders in the relationship between these measures. Future prospective studies with larger sample sizes and broader variable inclusion will enhance external validity and deepen understanding of these complex relationships.

## 5 Conclusion

Improving HRQOL remains a paramount goal in cancer care. This study has demonstrated that lower or decreasing SMI is associated with poorer HRQOL in the six months following diagnosis of upper gastrointestinal cancer. Malnutrition risk was a strong independent predictor of HRQOL at all timepoints and was also independently associated with SMI decrease over time, suggesting that malnutrition risk tools may offer a quick, low-cost way to identify patients at risk of skeletal muscle loss. As malnutrition risk is often modifiable, further research should focus on interventions that effectively reduce malnutrition risk and adequately control nutrition impact symptoms, which are likely to be central to concurrent improvement in HRQOL and attenuation of skeletal muscle loss in patients with cancer.

## CRediT authorship contribution statement

**LH**: Conceptualisation, Methodology, Formal Analysis, Investigation, Data Curation, Writing – Original Draft, Visualisation. **KN**: Writing – Review & Editing, Supervision. **JP**: Writing – Review & Editing, Supervision. **DC**: Investigation, Writing – Review & Editing, Supervision. **CEH**: Conceptualisation, Methodology, Investigation, Resources, Writing – Review & Editing, Supervision, Project administration, Funding Acquisition.

## Ethics approval and consent to participate

This study was approved by the Monash Health Human Research Ethics committee (RES-16-0000434A) and has therefore been performed in accordance with the ethical standards laid down in the 1964 Declaration of Helsinki and its later amendments. All participants provided informed consent prior to their inclusion in the study.

## Declaration of Generative AI and AI-assisted technologies in the writing process

Not applicable.

## Funding

This research was supported by an Australian Government Research Training Program (RTP) Scholarship, and a Monash University Postgraduate Publication award, to L.H. This work was also supported by a Victorian Cancer Agency Research Grant (grant number HSR15007, to C.E.H.) and a National Health and Medical Research Council (NHMRC) Translating Research Into Practice (TRIP) Fellowship (grant number GNT1168483, to C.E.H.).

## Availability of data and materials

Data described in the manuscript, code book and analytic code will be made available upon reasonable request pending application and ethical approval.

## Declaration of competing interest

The authors declare that they have no competing interests.
