# Supplementary material for: Malnutrition risk as a predictor of quality of life and skeletal muscle depletion following upper gastrointestinal cancer diagnosis: A longitudinal analysis
Source: J Nutr Health Aging. 2025 Jul 1;29(9):100623. doi: 10.1016/j.jnha.2025.100623 (PMC12270060; doi:10.1016/j.jnha.2025.100623)
Supplement: Supplementary file 1 [file mmc1.docx]

**Appendix A**

**Table A1. Differences in baseline characteristics between included participants, and those excluded due to absence of computed tomography imaging for analysis.**

|  |  | Included  *n* = 105 | Excluded  *n* = 6 | *p-*value |
| --- | --- | --- | --- | --- |
| ***Demographics*** |  |  |  |  |
| Age (years) |  |  |  |  |
|  |  | 65.9 (10.0) | 73.3 (8.8) | 0.076 |
| Sex, *n* (%) |  |  |  |  |
| Male |  | 71 (67.6) | 3 (50) | 0.398 |
| Female |  | 34 (32.4) | 3 (50) |  |
| Cancer type, *n* (%) |  |  |  |  |
| Oesophageal |  | 45 (42.9) | 1 (16.7) | 0.108 |
| Gastric |  | 21 (20.0) | 0 |  |
| Pancreatic |  | 39 (37.1) | 5 (83.3) |  |
| Clinical stage, *n* (%) |  |  |  |  |
| Resectable |  | 46 (43.8) | 3 (50) | 1.000 |
| Borderline resectable |  | 6 (5.7) | 2 (33.3) |  |
| Locally advanced |  | 31 (29.5) | 0 |  |
| Metastatic |  | 22 (21.0) | 1 (16.7) |  |
| BMI (kg/m^2^)^a^ |  | 25.1 (22.4-28.0) | 24.6 (21.8-26.8) | 0.620 |
|  |  |  |  |  |
| ***Malnutrition screening*** |  |  |  |  |
| PG-SGA_SF_ score |  | 8 (2-14) | 11 (4-16) | 0.460 |
|  |  |  |  |  |
| PG-SGA_SF_ category, n (%) |  |  |  |  |
| Score 0-1 |  | 20 (19.0) | 1 (16.7) | 0.928 |
| Score 2-3 |  | 14 (13.3) | - |  |
| Score 4-8 |  | 20 (19.0) | 1 (16.7) |  |
| Score ≥9 |  | 51 (48.6) | 4 (66.7) |  |
| ***Health-related quality of life*** |  |  |  |  |
| EORTC QLQ-C30 score^a^ |  |  |  |  |
| Global score |  | 66.7 (41.7-83.3) | 64.6 (45.8-77.1) | 0.922 |
| Physical function |  | 86.7 (66.7-100) | 80.0 (51.7-86.7) | 0.144 |
| Role function |  | 83.3 (33.3-100) | 41.7 (25-70.8) | 0.086 |
| Cognitive function |  | 83.3 (66.7-100) | 50.0 (29.2-87.5) | 0.023* |
| Social function |  | 83.3 (50-100) | 83.3 (50-83.3) | 0.419 |
| Emotional function |  | 75.0 (58.3-91.7) | 66.7 (54.2-79.2) | 0.343 |
| Summary score |  | 81.1 (66.0-91.5) | 70.0 (56.9-76.0) | 0.078 |

BMI body mass index; EORTC QLQ-C30 European Organisation for Research and Treatment of Cancer Quality of Life Questionnaire – Core 30; PG-SGA_SF_ Patient Generated Subjective Global Assessment Short Form; all continuous data presented as mean (standard deviation) unless otherwise indicated; ^a^data presented as median (IQR); **p*<0.05
