# Supplementary material for: Malnutrition risk as a predictor of quality of life and skeletal muscle depletion following upper gastrointestinal cancer diagnosis: A longitudinal analysis
Source: J Nutr Health Aging. 2025 Jul 1;29(9):100623. doi: 10.1016/j.jnha.2025.100623 (PMC12270060; doi:10.1016/j.jnha.2025.100623)
Supplement: Supplementary file 2 [file mmc2.docx]

**Appendix B**

**Contribution of skeletal muscle index and malnutrition risk to variation in EORTC QLQ-C30 scores following diagnosis of upper gastrointestinal cancer**

Low skeletal muscle index (SMI) and low skeletal muscle radiodensity (SMD) determined using cut points by Martin *et al* 2013 [1]

## Baseline (n=105)

**Table B1. EORTC QLQ-C30 Global Score - Baseline**

|  | Univariate analysis  β (95% CI) | p-value | Multivariate analysis  β (95% CI) | p-value | Multivariate analysis  β (95% CI) | p-value |
| --- | --- | --- | --- | --- | --- | --- |
|  |  |  | SMI (continuous) |  | Low (vs normal) SMI |  |
| SMI | 0.133 (-0.151, 0.807) | 0.178 | -0.138 (-0.907, 0.222) | 0.232^b^ |  |  |
| Low SMI | -0.132 (-16.843, 3.177) | 0.179 |  |  | -0.009 (-9.794, 8.909) | 0.925^b^ |
| PG-SGA_SF_ score^a^ |  |  |  |  |  |  |
| ≥ 2 | -0.397 (-37.622, -14.198) | <0.001 | -0.386 (-37.759, -12.621) | <0.001^c^ | -0.356 (-35.338, -11.101) | <0.001^d^ |
| ≥ 4 | -0.439 (-33.665, -14.423) | <0.001 | -0.439 (-35.130, -12.959) | <0.001^c^ | -0.391 (-31.947, -10.917) | <0.001^d^ |
| ≥ 9 | -0.468 (-32.859, -15.139) | <0.001 | -0.422 (-30.955, -12.350) | <0.001^c^ | -0.412 (-30.307, -11.963) | <0.001^d^ |

*n* = 105; EORTC QLQ-C30 European Organisation for Research and Treatment of Cancer Quality of Life Questionnaire – Core 30; PG-SGA_SF_ Patient Generated Subjective Global Assessment Short Form; SMI skeletal muscle index; ^a^PG-SGA_SF_ categories analysed independently; ^b^model 1 adjusted for age, sex, cancer type and baseline PG-SGA_SF_ score; ^c^model 2 adjusted for age, sex, cancer type and SMI (cm^2^/m^2^); ^d^model 3 adjusted for age, sex, cancer type and low (vs normal) SMI

**Table B2. EORTC QLQ-C30 Physical Function Score - Baseline**

|  | Univariate analysis  β (95% CI) | p-value | Multivariate analysis  β (95% CI) | p-value | Multivariate analysis  β (95% CI) | p-value |
| --- | --- | --- | --- | --- | --- | --- |
|  |  |  | SMI (continuous) |  | Low (vs normal) SMI |  |
| SMI | 0.205 (0.030, 0.852) | 0.036 | -0.043 (-0.595, 0.412) | 0.720^b^ |  |  |
| Low SMI | -0.140 (-14.946, 2.404) | 0.155 |  |  | 0.016 (-7.581, 8.995) | 0.866^b^ |
| PG-SGA_SF_ score^a^ |  |  |  |  |  |  |
| ≥ 2 | -0.302 (-27.670, -6.566) | 0.002 | -0.274 (-26.914, -4.078) | 0.008^c^ | -0.280 (-26.893, -4.884) | 0.005^d^ |
| ≥ 4 | -0.348 (-25.231, -7.810) | <0.001 | -0.329 (-25.727, -5.560) | 0.003^c^ | -0.328 (-25.119, -6.071) | 0.002^d^ |
| ≥ 9 | -0.426 (-26.817, -11.077) | <0.001 | -0.383 (-25.344, -8.760) | <0.001^c^ | -0.389 (-25.540, -9.115) | <0.00^d^ |

*n* = 105; EORTC QLQ-C30 European Organisation for Research and Treatment of Cancer Quality of Life Questionnaire – Core 30; PG-SGA_SF_ Patient Generated Subjective Global Assessment Short Form; SMI skeletal muscle index; ^a^PG-SGA_SF_ categories analysed independently; ^b^model 1 adjusted for age, sex, cancer type and baseline PG-SGA_SF_ score; ^c^model 2 adjusted for age, sex, cancer type and SMI (cm^2^/m^2^); ^d^model 3 adjusted for age, sex, cancer type and low (vs normal) SMI

**Table B3. EORTC QLQ-C30 Role Function Score - Baseline**

|  | Univariate analysis  β (95% CI) | p-value | Multivariate analysis  β (95% CI) | p-value | Multivariate analysis  β (95% CI) | p-value |
| --- | --- | --- | --- | --- | --- | --- |
|  |  |  | SMI (continuous) |  | Low (vs normal) SMI |  |
| SMI | 0.252 (0.213, 1.489) | 0.009 | -0.079 (-0.943, 0.412) | 0.439^b^ |  |  |
| Low SMI | -0.183 (-26.433, 0.641) | 0.062 |  |  | -0.013 (-12.124, 10.227) | 0.867^b^ |
| PG-SGA_SF_ score^a^ |  |  |  |  |  |  |
| ≥ 2 | -0.358 (-48.154, -15.670) | <0.001 | -0.305 (-43.315, -10.990) | 0.001^c^ | -0.296 (-41.877, -10.815) | 0.001^d^ |
| ≥ 4 | -0.466 (-47.717, -21.877) | <0.001 | -0.411 (-44.645, -16.802) | <0.001^c^ | -0.385 (-41.935, -15.644) | <0.001^d^ |
| ≥ 9 | -0.527 (-48.473, -25.238) | <0.001 | -0.437 (-41.983, -19.218) | <0.001^c^ | -0.435 (-41.664, -19.226) | <0.001^d^ |

*n* = 105; EORTC QLQ-C30 European Organisation for Research and Treatment of Cancer Quality of Life Questionnaire – Core 30; PG-SGA_SF_ Patient Generated Subjective Global Assessment Short Form; SMI skeletal muscle index; ^a^PG-SGA_SF_ categories analysed independently; ^b^model 1 adjusted for age, sex, cancer type and baseline PG-SGA_SF_ score; ^c^model 2 adjusted for age, sex, cancer type and SMI (cm^2^/m^2^); ^d^model 3 adjusted for age, sex, cancer type and low (vs normal) SMI

**Table B4. EORTC QLQ-C30 Social Function Score - Baseline**

|  | Univariate analysis  β (95% CI) | p-value | Multivariate analysis  β (95% CI) | p-value | Multivariate analysis  β (95% CI) | p-value |
| --- | --- | --- | --- | --- | --- | --- |
|  |  |  | SMI (continuous) |  | Low (vs normal) SMI |  |
| SMI | 0.078 (-0.345, 0.807) | 0.428 | -0.137 (-1.088, 0.280) | 0.244^b^ |  |  |
| Low SMI | -0.068 (-16.214, 7.863) | 0.493 |  |  | 0.022 (-9.980, 12.671) | 0.814^b^ |
| PG-SGA_SF_ score^a^ |  |  |  |  |  |  |
| ≥ 2 | -0.282 (-36.636, -7.383) | 0.004 | -0.235 (-34.014, -2.716) | 0.022^c^ | -0.221 (-32.311, -2.178) | 0.025^d^ |
| ≥ 4 | -0.410 (-38.525, -15.189) | <0.001 | -0.379 (-38.297, -11.379) | <0.001^c^ | -0.344 (-35.247, -9.737) | <0.001^d^ |
| ≥ 9 | -0.481 (-40.002, -19.003) | <0.001 | -0.434 (-37.578, -15.659) | <0.001^c^ | -0.427 (-36.982, -15.313) | <0.001^d^ |

*n* = 105; EORTC QLQ-C30 European Organisation for Research and Treatment of Cancer Quality of Life Questionnaire – Core 30; PG-SGA_SF_ Patient Generated Subjective Global Assessment Short Form; SMI skeletal muscle index; ^a^PG-SGA_SF_ categories analysed independently; ^b^model 1 adjusted for age, sex, cancer type and baseline PG-SGA_SF_ score; ^c^model 2 adjusted for age, sex, cancer type and SMI (cm^2^/m^2^); ^d^model 3 adjusted for age, sex, cancer type and low (vs normal) SMI

**Table B5. EORTC QLQ-C30 Cognitive Function Score - Baseline**

|  | Univariate analysis  β (95% CI) | p-value | Multivariate analysis  β (95% CI) | p-value | Multivariate analysis  β (95% CI) | p-value |
| --- | --- | --- | --- | --- | --- | --- |
|  |  |  | SMI (continuous) |  | Low (vs normal) SMI |  |
| SMI | -0.014 (-0.399, 0.347) | 0.891 | -0.370 (-1.166, -0.248) | 0.003^b^ |  |  |
| Low SMI | 0.032 (-6.500, 9.079) | 0.328 |  |  | 0.172 (-0.925, 14.634) | 0.084^b^ |
| PG-SGA_SF_ score^a^ |  |  |  |  |  |  |
| ≥ 2 | -0.266 (-22.876, -3.889) | 0.006 | -0.315 (-25.970, -5.781) | 0.002^c^ | -0.265 (-23.227, -3.441) | 0.009^d^ |
| ≥ 4 | -0.360 (-22.933, -7.514) | <0.001 | -0.445 (-27.488, -10.165) | <0.001^c^ | -0.362 (-23.749, -6.879) | <0.001^d^ |
| ≥ 9 | -0.465 (-25.275, -11.580) | <0.001 | -0.470 (-25.716, -11.507) | <0.001^c^ | -0.445 (-24.785, -10.479) | <0.001^d^ |

*n* = 105; EORTC QLQ-C30 European Organisation for Research and Treatment of Cancer Quality of Life Questionnaire – Core 30; PG-SGA_SF_ Patient Generated Subjective Global Assessment Short Form; SMI skeletal muscle index; ^a^PG-SGA_SF_ categories analysed independently; ^b^model 1 adjusted for age, sex, cancer type and baseline PG-SGA_SF_ score; ^c^model 2 adjusted for age, sex, cancer type and SMI (cm^2^/m^2^); ^d^model 3 adjusted for age, sex, cancer type and low (vs normal) SMI

**Table B6. EORTC QLQ-C30 Emotional Function Score - Baseline**

|  | Univariate analysis  β (95% CI) | p-value | Multivariate analysis  β (95% CI) | p-value | Multivariate analysis  β (95% CI) | p-value |
| --- | --- | --- | --- | --- | --- | --- |
|  |  |  | SMI (continuous) |  | Low (vs normal) SMI |  |
| SMI | 0.048 (-0.320, 0.531) | 0.624 | 0.025 (-0.511, 0.620) | 0.849^b^ |  |  |
| Low SMI | -0.091 (-13.014, 4.699) | 0.354 |  |  | -0.092 (-13.454, 5.084) | 0.373^b^ |
| PG-SGA_SF_ score^a^ |  |  |  |  |  |  |
| ≥ 2 | -0.228 (-24.054, -2.171) | 0.019 | -0.182 (-22.212, 1.309) | 0.081^c^ | -0.176 (-21.388, 1.163) | 0.078^d^ |
| ≥ 4 | -0.308 (-23.837, -5.893) | 0.001 | -0.272 (-23.478, -2.817) | 0.013^c^ | -0.255 (-22.021, -2.587) | 0.014^d^ |
| ≥ 9 | -0.271 (-20.727, -3.728) | 0.005 | -0.239 (-19.601, -2.023) | 0.016^c^ | -0.238 (-19.413, -2.113) | 0.015^d^ |

*n* = 105; EORTC QLQ-C30 European Organisation for Research and Treatment of Cancer Quality of Life Questionnaire – Core 30; PG-SGA_SF_ Patient Generated Subjective Global Assessment Short Form; SMI skeletal muscle index; ^a^Model 1 adjusted for age, sex, cancer type and baseline PG-SGA_SF_ score; ^b^Model 2 adjusted for age, sex, cancer type and SMI (cm^2^/m^2^); ^c^Model 3 adjusted for age, sex, cancer type and low (vs normal) SMI

**Table B7. EORTC QLQ-C30 Summary [2] - Baseline**

|  | Univariate analysis  β (95% CI) | p-value | Multivariate analysis  β (95% CI) | p-value | Multivariate analysis  β (95% CI) | p-value |
| --- | --- | --- | --- | --- | --- | --- |
|  |  |  | SMI (continuous) |  | Low (vs normal) SMI |  |
| SMI | 0.213 (0.038, 0.692) | 0.029 | -0.073 (-0.469, 0.218) | 0.471^b^ |  |  |
| Low SMI | -0.170 (-12.951, 0.820) | 0.084 |  |  | -0.018 (-6.303, 5.027) | 0.824^b^ |
| PG-SGA_SF_ score^a^ |  |  |  |  |  |  |
| ≥ 2 | -0.367 (-24.786, -8.361) | <0.001 | -0.312 (-22.764, -5.395) | 0.002^c^ | -0.308 (-22.252, -5.570) | 0.001^d^ |
| ≥ 4 | -0.499 (-25.338, -12.498) | <0.001 | -0.461 (-24.821, -10.096) | <0.001^c^ | -0.437 (-23.507, -9.617) | <0.001^d^ |
| ≥ 9 | -0.588 (-26.494, -15.278) | <0.001 | -0.525 (-24.461, -12.796) | <0.001^c^ | -0.524 (-24.342, -12.848) | <0.001^d^ |

*n* = 105; EORTC QLQ-C30 European Organisation for Research and Treatment of Cancer Quality of Life Questionnaire – Core 30; PG-SGA_SF_ Patient Generated Subjective Global Assessment Short Form; SMI skeletal muscle index; ^a^PG-SGA_SF_ categories analysed independently; ^b^model 1 adjusted for age, sex, cancer type and baseline PG-SGA_SF_ score; ^c^model 2 adjusted for age, sex, cancer type and SMI (cm^2^/m^2^); ^d^model 3 adjusted for age, sex, cancer type and low (vs normal) SMI

## Three months (n=65)

**Table B8. EORTC QLQ-C30 Global Score – Three months**

|  | Univariate analysis  β (95% CI) | p-value | Multivariate analysis  β (95% CI) | p-value | Multivariate analysis  β (95% CI) | p-value | Multivariate analysis  β (95% CI) | p-value |
| --- | --- | --- | --- | --- | --- | --- | --- | --- |
|  |  |  | SMI (continuous) |  | Low (vs normal) SMI |  | SMI decrease from baseline |  |
| SMI | 0.058 (-0.541, 0.862) | 0.649 | 0.005 (-0.824, 0.854) | 0.972^b,c^ |  |  |  |  |
| Low SMI | -0.031 (-14.433, 11.344) | 0.811 |  |  | 0.013 (-11.283, 12.611) | 0.912^b,c^ |  |  |
| SMI decrease from baseline^a^ | |  |  |  |  |  |  |  |
| ≥2% | -0.247 (-27.039, 0.068) | 0.051 |  |  |  |  | -0.227 (-26.321, 1.529) | 0.080^f^ |
| ≥5% | -0.350 (-28.918, -5.418) | 0.005 |  |  |  |  | -0.311 (-26.765, -3.680) | 0.011^f^ |
| ≥10% | -0.316 (-31.114, -4.047) | 0.012 |  |  |  |  | -0.288 (-29.508, -2.591) | 0.020^f^ |
| ≥20% | 0.001 (-35.239, 35.581) | 0.992 |  |  |  |  | -0.029 (-39.276, 31.363) | 0.823^f^ |
| PG-SGA_SF_ score^a,b^ | |  |  |  |  |  |  |  |
| ≥ 2 | -0.434 (-41.041, -12.554) | <0.001 | -0.434 (-42.354, -11.299) | 0.001^d^ | -0.434 (-42.299, -11.253) | 0.001^e^ | -0.379 (-38.310, -8.461) | 0.003^g^ |
| ≥ 4 | -0.542 (-40.018, -17.288) | <0.001 | -0.577 (-43.690, -17.322) | <0.001^d^ | -0.571 (-43.346, -17.035) | <0.001^e^ | -0.510 (-39.776, -14.128) | <0.001^g^ |
| ≥ 9 | -0.451 (-32.973, -10.811) | <0.001 | -0.440 (-33.688, -9.030) | 0.001^d^ | -0.441 (-33.698, -9.040) | <0.001^e^ | -0.361 (-29.862, -5.197) | 0.006^g^ |

*n*=65; EORTC QLQ-C30 European Organisation for Research and Treatment of Cancer Quality of Life Questionnaire – Core 30; PG-SGA_SF_ Patient Generated Subjective Global Assessment Short Form; SMI skeletal muscle index; ^a^categories analysed independently; ^b^n=64, data missing for one participant due to not completing PG-SGA_SF_ form; ^c^model 1 adjusted for age, sex, cancer type and three-month PG-SGA_SF_ score; ^d^model 2 adjusted for age, sex, cancer type and SMI at three months (cm^2^/m^2^); ^e^model 3 adjusted for age, sex, cancer type and low (vs normal) SMI at three months; ^f^model 4 adjusted for age, sex and cancer type and baseline PG-SGA_SF_ score; ^g^model 5 adjusted for age, sex and cancer type and percentage SMI decrease from baseline

**Table B9. EORTC QLQ-C30 Physical Function Score – Three months**

|  | Univariate analysis  β (95% CI) | p-value | Multivariate analysis  β (95% CI) | p-value | Multivariate analysis  β (95% CI) | p-value | Multivariate analysis  β (95% CI) | p-value |
| --- | --- | --- | --- | --- | --- | --- | --- | --- |
|  |  |  | SMI (continuous) |  | Low (vs normal) SMI |  | SMI decrease from baseline |  |
| SMI | 0.202 (-0.112, 1.140) | 0.106 | 0.193 (-0.257, 1.235) | 0.194^b,c^ |  |  |  |  |
| Low SMI | -0.218 (-21.530, 1.268) | 0.081 |  |  | -0.203 (-19.890, 1.007) | 0.076^b,c^ |  |  |
| SMI decrease from baseline^a^ | |  |  |  |  |  |  |  |
| ≥2% | -0.206 (-22.404, 2.026) | 0.101 |  |  |  |  | -0.235 (-23.503, 0.229) | 0.054^f^ |
| ≥5% | -0.152 (-17.956, 4.340) | 0.227 |  |  |  |  | -0.124 (-15.985, 4.861) | 0.290^f^ |
| ≥10% | -0.214 (-23.710, 1.618) | 0.086 |  |  |  |  | -0.181 (-21.238, 2.645)) | 0.125^f^ |
| ≥20% | -0.084 (-42.970, 21.488) | 0.508 |  |  |  |  | -0.123 (-46.148, 14.451) | 0.300^f^ |
| PG-SGA_SF_ score^a,b^ | |  |  |  |  |  |  |  |
| ≥ 2 | -0.384 (-.34.176, -8.388) | 0.002 | -0.367 (-33.616, -7.115_ | 0.003^d^ | -0.363 (-33.202, -7.050) | 0.003^e^ | -0.316 (-30.761, -4.282) | 0.010^g^ |
| ≥ 4 | -0.521 (-34.912, -14.525) | <0.001 | -0.511 (-35.594, -12.929) | <0.001^d^ | -0.501 (-34.917, -12.571) | <0.001^e^ | -0.444 (-32.627, -9.457) | <0.001^g^ |
| ≥ 9 | -0.424 (-29.231, -8.686) | <0.001 | -0.349 (-26.547, -4.674) | 0.006^d^ | -0.350 (-26.420, -4.820) | 0.005^e^ | -0.299 (-24.702, -2.057) | 0.021^g^ |

*n*=65; EORTC QLQ-C30 European Organisation for Research and Treatment of Cancer Quality of Life Questionnaire – Core 30; PG-SGA_SF_ Patient Generated Subjective Global Assessment Short Form; SMI skeletal muscle index; ^a^categories analysed independently; ^b^n=64, data missing for one participant due to not completing PG-SGA_SF_ form; ^c^model 1 adjusted for age, sex, cancer type and three-month PG-SGA_SF_ score; ^d^model 2 adjusted for age, sex, cancer type and SMI at three months (cm^2^/m^2^); ^e^model 3 adjusted for age, sex, cancer type and low (vs normal) SMI at three months; ^f^model 4 adjusted for age, sex and cancer type and baseline PG-SGA_SF_ score; ^g^model 5 adjusted for age, sex and cancer type and percentage SMI decrease from baseline

**Table B10. EORTC QLQ-C30 Role Function Score – Three months**

|  | Univariate analysis  β (95% CI) | p-value | Multivariate analysis  β (95% CI) | p-value | Multivariate analysis  β (95% CI) | p-value | Multivariate analysis  β (95% CI) | p-value |
| --- | --- | --- | --- | --- | --- | --- | --- | --- |
|  |  |  | SMI (continuous) |  | Low (vs normal) SMI |  | SMI decrease from baseline |  |
| SMI | -0.052 (-1.156, 0.761) | 0.682 | -0.051 (-1.433, 1.046) | 0.756^b,c^ |  |  |  |  |
| Low SMI | 0.003 (-17.313, 17.762) | 0.980 |  |  | -0.012 (-18.465, 16.725) | 0.922^b,c^ |  |  |
| SMI decrease from baseline^a^ | |  |  |  |  |  |  |  |
| ≥2% | -0.323 (-41.807, -6.341) | 0.009 |  |  |  |  | -0.314 (-42.610, -4.186) | 0.018^f^ |
| ≥5% | -0.386 (-41.559, -10.308) | 0.002 |  |  |  |  | -0.362 (-40.479, -8.252) | 0.004^f^ |
| ≥10% | -0.347 (-45.067, -8.547) | 0.005 |  |  |  |  | -0.316 (-43.441, -5.409) | 0.013^f^ |
| ≥20% | 0.009 (-46.838, 50.277) | 0.944 |  |  |  |  | -0.017 (-53.530, 47.109) | 0.899^f^ |
| PG-SGA_SF_ score^a,b^ | |  |  |  |  |  |  |  |
| ≥2 | -0.323 (-7.085, -46.761) | 0.009 | -0.332 (-49.215, -6.047) | 0.013^d^ | -0.334 (-49.368, -6.329) | 0.012^e^ | -0.268 (-42.245, -2.426) | 0.029^g^ |
| ≥4 | -0.421 (-46.276, -13.753) | <0.001 | -0.466 (-51.998, -14.421) | <0.001^d^ | -0.467 (-51.963, -14.620) | <0.001^e^ | -0.381 (-44.755, -9.520) | 0.003^g^ |
| ≥9 | -0.318 (-37.500, -5.209) | 0.010 | -0.320 (-39.237, -3.754) | 0.018^d^ | -0.319 (-39.124, -3.621) | 0.019^e^ | -0.205 (-30.926, 3.402) | 0.114^g^ |

*n*=65; EORTC QLQ-C30 European Organisation for Research and Treatment of Cancer Quality of Life Questionnaire – Core 30; PG-SGA_SF_ Patient Generated Subjective Global Assessment Short Form; SMI skeletal muscle index; ^a^categories analysed independently; ^b^n=64, data missing for one participant due to not completing PG-SGA_SF_ form; ^c^model 1 adjusted for age, sex, cancer type and three-month PG-SGA_SF_ score; ^d^model 2 adjusted for age, sex, cancer type and SMI at three months (cm^2^/m^2^); ^e^model 3 adjusted for age, sex, cancer type and low (vs normal) SMI at three months; ^f^model 4 adjusted for age, sex and cancer type and baseline PG-SGA_SF_ score; ^g^model 5 adjusted for age, sex and cancer type and percentage SMI decrease from baseline

**Table B11. EORTC QLQ-C30 Social Function Score – Three months**

|  | Univariate analysis  β (95% CI) | p-value | Multivariate analysis  β (95% CI) | p-value | Multivariate analysis  β (95% CI) | p-value | Multivariate analysis  β (95% CI) | p-value |
| --- | --- | --- | --- | --- | --- | --- | --- | --- |
|  |  |  | SMI (continuous) |  | Low (vs normal) SMI |  | SMI decrease from baseline |  |
| SMI | 0.126 (-0.500, 1.481) | 0.126 | 0.134 (-0.761, 1.804) | 0.419^b,c^ |  |  |  |  |
| Low SMI | -0.109 (-25.992, 10.449) | 0.397 |  |  | -0.079 (-23.972, 12.648) | 0.538^b,c^ |  |  |
| SMI decrease from baseline^a^ | |  |  |  |  |  |  |  |
| ≥2% | -0.352 (-45.970, -8.761) | 0.005 |  |  |  |  | -0.396 (-50.800, -10.630) | 0.003^f^ |
| ≥5% | -0.379 (-42.891, -9.887) | 0.002 |  |  |  |  | -0.378 (-43.485, -9.171) | 0.003^f^ |
| ≥10% | -0.327 (-45.025, -6.703) | 0.009 |  |  |  |  | -0.343 (-47.295, -7.029) | 0.009^f^ |
| ≥20% | 0.167 (-16.840, 82.414) | 0.191 |  |  |  |  | 0.162 (-20.990, 84.722) | 0.232^f^ |
| PG-SGA_SF_ score^a,b^ | |  |  |  |  |  |  |  |
| ≥2 | -0.232 (-42.204, 1.517) | 0.068 | -0.239 (-44.857, 2.936) | 0.084^d^ | -0.236 (-44.663, 3.189) | 0.088^e^ | -0.169 (-37.722, 8.008) | 0.198^g^ |
| ≥4 | -0.354 (-44.594, -8.635) | 0.004 | -0.406 (-51.154, -9.753) | 0.005^d^ | -0.393 (-50.256, -8.822) | 0.006^e^ | -0.317 (-43.918, -3.703) | 0.021^g^ |
| ≥9 | -0.354 (-40.904, -7.886) | 0.004 | -0.364 (-43.424, -6.792) | 0.008^d^ | -0.365 (-43.471, -6.788) | 0.008^e^ | -0.276 (-37.271, -0.803) | 0.041^g^ |

*n*=65; EORTC QLQ-C30 European Organisation for Research and Treatment of Cancer Quality of Life Questionnaire – Core 30; PG-SGA_SF_ Patient Generated Subjective Global Assessment Short Form; SMI skeletal muscle index; ^a^categories analysed independently; ^b^n=64, data missing for one participant due to not completing PG-SGA_SF_ form; ^c^model 1 adjusted for age, sex, cancer type and three-month PG-SGA_SF_ score; ^d^model 2 adjusted for age, sex, cancer type and SMI at three months (cm^2^/m^2^); ^e^model 3 adjusted for age, sex, cancer type and low (vs normal) SMI at three months; ^f^model 4 adjusted for age, sex and cancer type and baseline PG-SGA_SF_ score; ^g^model 5 adjusted for age, sex and cancer type and percentage SMI decrease from baseline

**Table B12. EORTC QLQ-C30 Cognitive Function Score – Three months**

|  | Univariate analysis  β (95% CI) | p-value | Multivariate analysis  β (95% CI) | p-value | Multivariate analysis  β (95% CI) | p-value | Multivariate analysis  β (95% CI) | p-value |
| --- | --- | --- | --- | --- | --- | --- | --- | --- |
|  |  |  | SMI (continuous) |  | Low (vs normal) SMI |  | SMI decrease from baseline |  |
| SMI | 0.070 (-0.496, 0.871) | 0.586 | -0.014 (-0.948, 0.874) | 0.936^b,c^ |  |  |  |  |
| Low SMI | 0.006 (-12.305, 12.848) | 0.966 |  |  | 0.060 (-10.011, 15.897) | 0.651^b,c^ |  |  |
| SMI decrease from baseline^a^ | |  |  |  |  |  |  |  |
| ≥2% | -0.188 (-23.435, 3.359) | 0.139 |  |  |  |  | -0.205 (-25.150, 3.295) | 0.129^f^ |
| ≥5% | -0.187 (-20.970, 3.069) | 0.142 |  |  |  |  | -0.173 (-20.487, 3.931) | 0.180^f^ |
| ≥10% | -0.280 (-28.579, -1.873) | 0.026 |  |  |  |  | -0.300 (-29.962, -2.660) | 0.020^f^ |
| ≥20% | -0.015 (-36.582, 32.483) | 0.906 |  |  |  |  | -0.070 (-45.252, 26.264) | 0.597^f^ |
| PG-SGA_SF_ score^a,b^ | |  |  |  |  |  |  |  |
| ≥2 | -0.159, (-24.783, 5.666) | 0.214 | -0.160 (-26.342, 7.032) | 0.251^d^ | -0.159 (-26.252, 7.074) | 0.254^e^ | -0.108 (-22.792, 9.735) | 0.425^g^ |
| ≥4 | -0.274 (-26.804, -1.426) | 0.030 | -0.294 (-29.891, -0.385) | 0.045^d^ | -0.291 (-29.658, -0.300) | 0.046^e^ | -0.232 (-26.489, 2.539) | 0.104^g^ |
| ≥9 | -0.287 (-25.162, -1.955) | 0.023 | -0.288 (-26.523, -0.692) | 0.039^d^ | -0.289 (-26.577, -0.780) | 0.038^e^ | -0.214 (-23.305, 2.922) | 0.126^g^ |

*n*=65; EORTC QLQ-C30 European Organisation for Research and Treatment of Cancer Quality of Life Questionnaire – Core 30; PG-SGA_SF_ Patient Generated Subjective Global Assessment Short Form; SMI skeletal muscle index; ^a^categories analysed independently; ^b^n=64, data missing for one participant due to not completing PG-SGA_SF_ form; ^c^model 1 adjusted for age, sex, cancer type and three-month PG-SGA_SF_ score; ^d^model 2 adjusted for age, sex, cancer type and SMI at three months (cm^2^/m^2^); ^e^model 3 adjusted for age, sex, cancer type and low (vs normal) SMI at three months; ^f^model 4 adjusted for age, sex and cancer type and baseline PG-SGA_SF_ score; ^g^model 5 adjusted for age, sex and cancer type and percentage SMI decrease from baseline

**Table B13. EORTC QLQ-C30 Emotional Function Score – Three months**

|  | Univariate analysis  β (95% CI) | p-value | Multivariate analysis  β (95% CI) | p-value | Multivariate analysis  β (95% CI) | p-value | Multivariate analysis  β (95% CI) | p-value |
| --- | --- | --- | --- | --- | --- | --- | --- | --- |
|  |  |  | SMI (continuous) |  | Low (vs normal) SMI |  | SMI decrease from baseline |  |
| SMI | 0.119 (-0.332, 0.913) | 0.354 | -0.105 (-1.095, 0.580) | 0.540^b,c^ |  |  |  |  |
| Low SMI | 0.030 (-10.131, 12.867) | 0.813 |  |  | 0.155 (-4.870, 18.784) | 0.244^b,c^ |  |  |
| SMI decrease from baseline^a^ | |  |  |  |  |  |  |  |
| ≥2% | -0.094 (-16.985, 7.863) | 0.466 |  |  |  |  | -0.210 (-23.290, 2.779) | 0.121^f^ |
| ≥5% | -0.162 (-18.143, 3.945) | 0.204 |  |  |  |  | -0.219 (-20.686, 1.497) | 0.089^f^ |
| ≥10% | 0.115 (-6.945, 18.337) | 0.371 |  |  |  |  | 0.042 (-11.030, 15.220) | 0.750^f^ |
| ≥20% | 0.079 (-21.726, 41.261) | 0.537 |  |  |  |  | 0.067 (-24.560, 41.062) | 0.616^f^ |
| PG-SGA_SF_ score^a,b^ | |  |  |  |  |  |  |  |
| ≥2 | -0.103 (-19.708, 8.352) | 0.422 | -0.115 (-20.902, 8.269) | 0.389^d^ | -0.114 (-20.756, 8.176) | 0.388^e^ | -0.108 (-20.793, 8.849) | 0.423^g^ |
| ≥4 | -0.107 (-17.063, 6.935) | 0.402 | -0.144 (-19.960, 6.387) | 0.307^d^ | -0.147 (-19.940, 6.077) | 0.290^e^ | -0.141 (-20.058, 6.791) | 0.327^g^ |
| ≥9 | -0.094 (-15.096, 6.964) | 0.464 | -0.116 (-16.599, 6.573) | 0.390^d^ | -0.119 (-16.624, 6.367) | 0.375^e^ | -0.100 (-16.438, 7.762) | 0.476^g^ |

*n*=65; EORTC QLQ-C30 European Organisation for Research and Treatment of Cancer Quality of Life Questionnaire – Core 30; PG-SGA_SF_ Patient Generated Subjective Global Assessment Short Form; SMI skeletal muscle index; ^a^categories analysed independently; ^b^n=64, data missing for one participant due to not completing PG-SGA_SF_ form; ^c^model 1 adjusted for age, sex, cancer type and three-month PG-SGA_SF_ score; ^d^model 2 adjusted for age, sex, cancer type and SMI at three months (cm^2^/m^2^); ^e^model 3 adjusted for age, sex, cancer type and low (vs normal) SMI at three months; ^f^model 4 adjusted for age, sex and cancer type and baseline PG-SGA_SF_ score; ^g^model 5 adjusted for age, sex and cancer type and percentage SMI decrease from baseline

**Table B14. EORTC QLQ-C30 Summary Score [2] – Three months**

|  | Univariate analysis  β (95% CI) | p-value | Multivariate analysis  β (95% CI) | p-value | Multivariate analysis  β (95% CI) | p-value | Multivariate analysis  β (95% CI) | p-value |
| --- | --- | --- | --- | --- | --- | --- | --- | --- |
|  |  |  | SMI (continuous) |  | Low (vs normal) SMI |  | SMI decrease from baseline |  |
| SMI | 0.032 (-0.446, 0.573) | 0.804 | 0.032 (-0.5445, 0.672) | 0.835^b,c^ |  |  |  |  |
| Low SMI | 0.011 (-8.959, 9.743) | 0.934 |  |  | 0.034 (-7.412, 9.900) | 0.775^b,c^ |  |  |
| SMI decrease from baseline^a^ | |  |  |  |  |  |  |  |
| ≥2% | -0.296 (-21.427, -2.051) | 0.018 |  |  |  |  | -0.275 (-20.988, -0.773) | 0.035^f^ |
| ≥5% | -0.334 (-20.430, 3.276) | 0.008 |  |  |  |  | -0.297 (-19.104, -2.034) | 0.016^f^ |
| ≥10% | -0.377 (-24.794, -5.629) | 0.002 |  |  |  |  | -0.361 (-24.198, -4.953) | 0.004^f^ |
| ≥20% | 0.002 (-25.457, 25.904) | 0.956 |  |  |  |  | -0.051 (-31.037, 20.812) | 0.694^f^ |
| PG-SGA_SF_ score^a,b^ | |  |  |  |  |  |  |  |
| ≥2 | -0.354 (-26.593, -5.150) | 0.004 | -0.375 (-28.494, -5.081) | 0.006^d^ | -0.373 (-28.403, -4.979) | 0.006^e^ | -0.309 (-24.918, -2.754) | 0.015^g^ |
| ≥4 | -0.482 (-27.064, -9.873) | <0.001 | -0.550 (-30.933, -11.187) | <0.001^d^ | -0.541 (-30.607, -10.874) | <0.001^e^ | -0.470 (-27.465, -8.525) | <0.001^g^ |
| ≥9 | -0.370 (-21.392, -4.661) | 0.003 | -0.379 (-22.634, -4.035) | 0.006^d^ | -0.381 (-22.689, -4.094) | 0.006^e^ | -0.283 (-19.097, -0.803) | 0.034^g^ |

*n*=65; EORTC QLQ-C30 European Organisation for Research and Treatment of Cancer Quality of Life Questionnaire – Core 30; PG-SGA_SF_ Patient Generated Subjective Global Assessment Short Form; SMI skeletal muscle index; ^a^categories analysed independently; ^b^n=64, data missing for one participant due to not completing PG-SGA_SF_ form; ^c^model 1 adjusted for age, sex, cancer type and three-month PG-SGA_SF_ score; ^d^model 2 adjusted for age, sex, cancer type and SMI at three months (cm^2^/m^2^); ^e^model 3 adjusted for age, sex, cancer type and low (vs normal) SMI at three months; ^f^model 4 adjusted for age, sex and cancer type and baseline PG-SGA_SF_ score; ^g^model 5 adjusted for age, sex and cancer type and percentage SMI decrease from baseline

## Six months (n=48)

**Table B15. EORTC QLQ-C30 Global Score – Six months**

|  | Univariate analysis  β (95% CI) | p-value | Multivariate analysis  β (95% CI) | p-value | Multivariate analysis  β (95% CI) | p-value |
| --- | --- | --- | --- | --- | --- | --- |
|  |  |  | SMI (continuous) |  | Low (vs normal) SMI |  |
| SMI | 0.009 (-0.692, 0.733) | 0.954 | 0.115 (-0.539, 1.090) | 0.498^b^ |  |  |
| Low SMI | -0.095 (-16.126, 8.302) | 0.522 |  |  | -0.155 (-18.110, 5.281) | 0.275^b^ |
| PG-SGA_SF_ score^a^ |  |  |  |  |  |  |
| ≥ 2 | -0.275 (-31.779, 0.580) | 0.058 | -0.269 (-32.578, 2.117) | 0.084^c^ | -0.292 (-34.008, 0.850) | 0.062^d^ |
| ≥ 4 | -0.317 (-23.989, -1.439) | 0.028 | -0.335 (-25.600, -1.235) | 0.032^c^ | -0.353 (-26.247, -2.075) | 0.023^d^ |
| ≥ 9 | -0.417 (-30.249, -6.498) | 0.003 | -0.445 (-32.207, -6.993) | 0.003^c^ | -0.442 (-31.968, -6.920) | 0.003^d^ |

*n* = 48; EORTC QLQ-C30 European Organisation for Research and Treatment of Cancer Quality of Life Questionnaire – Core 30; PG-SGA_SF_ Patient Generated Subjective Global Assessment Short Form; SMI skeletal muscle index; ^a^PG-SGA_SF_ categories analysed independently; ^b^model 1 adjusted for age, sex and cancer type and PG-SGA_SF_ score at six months; ^c^model 2 adjusted for age, sex, cancer type and SMI (cm^2^/m^2^) at six months; ^d^model 3 adjusted for age, sex, cancer type and low (vs normal) SMI at six months

**Table B16. EORTC QLQ-C30 Physical Function Score – Six months**

|  | Univariate analysis  β (95% CI) | p-value | Multivariate analysis  β (95% CI) | p-value | Multivariate analysis  β (95% CI) | p-value |
| --- | --- | --- | --- | --- | --- | --- |
|  |  |  | SMI (continuous) |  | Low (vs normal) SMI |  |
| SMI | 0.368 (0.221, 1.544) | 0.010 | 0.373 (0.172, 1.616) | 0.017^b^ |  |  |
| Low SMI | -0.242 (-21.888, 1.888) | 0.097 |  |  | -0.212 (-19.641, 2.216) | 0.112^b^ |
| PG-SGA_SF_ score^a^ |  |  |  |  |  |  |
| ≥ 2 | -0.339 (-35.022, -3.399) | 0.018 | -0.398 (-37.163, -7.962) | 0.003^c^ | -0.421 (-38.957, -8.779) | 0.003^d^ |
| ≥ 4 | -0.277 (-22.483, 0.338) |  | -0.324 (-23.849, -2.093) | 0.021^c^ | -0.304 (-23.437, -0.862) | 0.036^d^ |
| ≥ 9 | -0.253 (-23.747, 1.506) |  | -0.371 (-27.865, -4.761) | 0.007^c^ | -0.352 (-27.409, -3.536) | 0.012^d^ |

*n* = 48; EORTC QLQ-C30 European Organisation for Research and Treatment of Cancer Quality of Life Questionnaire – Core 30; PG-SGA_SF_ Patient Generated Subjective Global Assessment Short Form; SMI skeletal muscle index; ^a^PG-SGA_SF_ categories analysed independently; ^b^model 1 adjusted for age, sex and cancer type and PG-SGA_SF_ score at six months; ^c^model 2 adjusted for age, sex, cancer type and SMI (cm^2^/m^2^) at six months; ^d^model 3 adjusted for age, sex, cancer type and low (vs normal) SMI at six months

**Table B17. EORTC QLQ-C30 Role Function Score – Six months**

|  | Univariate analysis  β (95% CI) | p-value | Multivariate analysis  β (95% CI) | p-value | Multivariate analysis  β (95% CI) | p-value |
| --- | --- | --- | --- | --- | --- | --- |
|  |  |  | SMI (continuous) |  | Low (vs normal) SMI |  |
| SMI | 0.108 (-0.661, 1.422) | 0.466 | 0.261 (-0.176, 2.019) | 0.098^b^ |  |  |
| Low SMI | -0.134 (-26.027, 9.731) | 0.364 |  |  | -0.197 (-27.972, 4.049) | 0.139^b^ |
| PG-SGA_SF_ score^a^ |  |  |  |  |  |  |
| ≥ 2 | -0.354 (-52.648, 6.353) | 0.014 | -0.356 (-54.009, -5.310) | 0.018^c^ | -0.380 (-56.258, -7.091) | 0.013^d^ |
| ≥ 4 | -0.352 (-37.117, -4.391) | 0.014 | -0.397 (-40.543, -6.201) | 0.009^c^ | -0.396 (-40.615, -6.049) | 0.009^d^ |
| ≥ 9 | -0.541 (-51.178, -18.850) | <0.001 | -0.587 (-54.499, -21.568) | <0.001^c^ | -0.575 (-53.856, -20.597) | <0.001^d^ |

*n* = 48; EORTC QLQ-C30 European Organisation for Research and Treatment of Cancer Quality of Life Questionnaire – Core 30; PG-SGA_SF_ Patient Generated Subjective Global Assessment Short Form; SMI skeletal muscle index; ^a^PG-SGA_SF_ categories analysed independently; ^b^model 1 adjusted for age, sex and cancer type and PG-SGA_SF_ score at six months; ^c^model 2 adjusted for age, sex, cancer type and SMI (cm^2^/m^2^) at six months; ^d^model 3 adjusted for age, sex, cancer type and low (vs normal) SMI at six months

**Table B18. EORTC QLQ-C30 Social Function Score – Six months**

|  | Univariate analysis  β (95% CI) | p-value | Multivariate analysis  β (95% CI) | p-value | Multivariate analysis  β (95% CI) | p-value |
| --- | --- | --- | --- | --- | --- | --- |
|  |  |  | SMI (continuous) |  | Low (vs normal) SMI |  |
| SMI | 0.268 (-0.053, 1.611) | 0.066 | 0.360 (-0.086, 2.009) | 0.033^b^ |  |  |
| Low SMI | -0.192 (-24.222, 4.963) | 0.191 |  |  | -0.228 (-25.712, 2.839) | 0.113^b^ |
| PG-SGA_SF_ score^a^ |  |  |  |  |  |  |
| ≥ 2 | -0.078 (-25.678, 14.992) | 0.599 | -0.128 (-29.798, 12.178) | 0.402^c^ | -0.147 (-31.660, 11.467) | 0.350^d^ |
| ≥ 4 | -0.189 (-23.311, 4.992) | 0.199 | -0.271 (-27.768, 1.416) | 0.076^c^ | -0.257 (-27.420, 2.428) | 0.098^d^ |
| ≥ 9 | -0.478 (-39.405, -11.576) | <0.001 | -0.553 (-43.214, -15.851) | <0.001^c^ | -0.534 (-42.634, 14.403) | <0.001^d^ |

*n* = 48; EORTC QLQ-C30 European Organisation for Research and Treatment of Cancer Quality of Life Questionnaire – Core 30; PG-SGA_SF_ Patient Generated Subjective Global Assessment Short Form; SMI skeletal muscle index; ^a^PG-SGA_SF_ categories analysed independently; ^b^model 1 adjusted for age, sex and cancer type and PG-SGA_SF_ score at six months; ^c^model 2 adjusted for age, sex, cancer type and SMI (cm^2^/m^2^) at six months; ^d^model 3 adjusted for age, sex, cancer type and low (vs normal) SMI at six months

**Table B19. EORTC QLQ-C30 Cognitive Function Score – Six months**

|  | Univariate analysis  β (95% CI) | p-value | Multivariate analysis  β (95% CI) | p-value | Multivariate analysis  β (95% CI) | p-value |
| --- | --- | --- | --- | --- | --- | --- |
|  |  |  | SMI (continuous) |  | Low (vs normal) SMI |  |
| SMI | 0.090 (-0.436, 0.817) | 0.543 | -0.093 (-0.944, 0.551) | 0.599^b^ |  |  |
| Low SMI | -0.030 (-11.935, 9.713) | 0.837 |  |  | -0.036 (-9.534, 12.184) | 0.807^b^ |
| PG-SGA_SF_ score^a^ |  |  |  |  |  |  |
| ≥ 2 | -0.182 (-23.777, 5.426) | 0.212 | -0.214 (-25.402, 3.972) | 0.148^c^ | -0.216 (-25.787, 4.149) | 0.152^d^ |
| ≥ 4 | -0.313 (-21.039, -1.106) | 0.030 | -0.293 (-20.642, -0.056) | 0.049^c^ | -0.304 (-21.094, -0.437) | 0.041^d^ |
| ≥ 9 | -0.148 (-17.149, 5.665) | 0.316 | -0.213 (-19.720, 3.166) | 0.152^c^ | -0.219 (-19.947, 2.930) | 0.141^d^ |

*n* = 48; EORTC QLQ-C30 European Organisation for Research and Treatment of Cancer Quality of Life Questionnaire – Core 30; PG-SGA_SF_ Patient Generated Subjective Global Assessment Short Form; SMI skeletal muscle index; ^a^PG-SGA_SF_ categories analysed independently; ^b^model 1 adjusted for age, sex and cancer type and PG-SGA_SF_ score at six months; ^c^model 2 adjusted for age, sex, cancer type and SMI (cm^2^/m^2^) at six months; ^d^model 3 adjusted for age, sex, cancer type and low (vs normal) SMI at six months

**Table B20. EORTC QLQ-C30 Emotional Function Score – Six months**

|  | Univariate analysis  β (95% CI) | p-value | Multivariate analysis  β (95% CI) | p-value | Multivariate analysis  β (95% CI) | p-value |
| --- | --- | --- | --- | --- | --- | --- |
|  |  |  | SMI (continuous) |  | Low (vs normal) SMI |  |
| SMI | 0.129 (-0.389, 0.995) | 0.383 | 0.182 (-0.353, 1.212) | 0.275^b^ |  |  |
| Low SMI | -0.222 (-20.704, 2.741) | 0.130 |  |  | -0.258 (-21.473, 0.601) | 0.063^b^ |
| PG-SGA_SF_ score^a^ |  |  |  |  |  |  |
| ≥ 2 | -0.199 (-27.223, 5.097) | 0.175 | -0.211 (-28.079, 4.673) | 0.157^c^ | -0.249 (-29.977, 2.354) | 0.092^d^ |
| ≥ 4 | -00265 (-21.626, 0.843) | 0.069 | -0.330 (-24.261, -1.626) | 0.026^c^ | -0.361 (-25.144, -3.168) | 0.013^d^ |
| ≥ 9 | -0.392 (-28.692, -5.132) | 0.006 | -0.404 (-29.298, -5.522) | 0.005^c^ | -0.397 (-28.774, -5.502) | 0.005^d^ |

*n* = 48; EORTC QLQ-C30 European Organisation for Research and Treatment of Cancer Quality of Life Questionnaire – Core 30; PG-SGA_SF_ Patient Generated Subjective Global Assessment Short Form; SMI skeletal muscle index; ^a^PG-SGA_SF_ categories analysed independently; ^b^model 1 adjusted for age, sex and cancer type and PG-SGA_SF_ score at six months; ^c^model 2 adjusted for age, sex, cancer type and SMI (cm^2^/m^2^) at six months; ^d^model 3 adjusted for age, sex, cancer type and low (vs normal) SMI at six months

**Table B21. EORTC QLQ-C30 Summary Score [2] – Six months**

|  | Univariate analysis  β (95% CI) | p-value | Multivariate analysis  β (95% CI) | p-value | Multivariate analysis  β (95% CI) | p-value |
| --- | --- | --- | --- | --- | --- | --- |
|  |  |  | SMI (continuous) |  | Low (vs normal) SMI |  |
| SMI | 0.142 (-0.238, 0.682) | 0.336 | 0.265 (-0.045, 0.874) | 0.076^b^ |  |  |
| Low SMI | -0.095 (-16.126, 8.302) | 0.522 |  |  | -0.250 (-13.335, -0.149) | 0.045^b^ |
| PG-SGA_SF_ score^a^ |  |  |  |  |  |  |
| ≥ 2 | -0.275 (-31.779, 0.580) | 0.058 | -0.435 (-26.487, -5.649) | 0.003^c^ | -0.470 (-27.714, -7.036) | 0.002^d^ |
| ≥ 4 | -0.317 (-23.989, -1.439) | 0.028 | -0.461 (-19.410, -4.649) | 0.002^c^ | -0.473 (-19.668, -5.054) | 0.001^d^ |
| ≥ 9 | -0.417 (-30.249, -6.498) | 0.003 | -0.621 (-24.911, -10.737) | <0.001)^c^ | -0.609 (-24.572, -10.420) | <0.001^d^ |

*n* = 48; EORTC QLQ-C30 European Organisation for Research and Treatment of Cancer Quality of Life Questionnaire – Core 30; PG-SGA_SF_ Patient Generated Subjective Global Assessment Short Form; SMI skeletal muscle index; ^a^PG-SGA_SF_ categories analysed independently; ^b^model 1 adjusted for age, sex and cancer type and PG-SGA_SF_ score; ^c^model 2 adjusted for age, sex, cancer type and SMI (cm^2^/m^2^); ^d^model 3 adjusted for age, sex, cancer type and low (vs normal) SMI

**References**

1. Martin L, Birdsell L, Macdonald N, Reiman T, Clandinin MT, McCargar LJ, et al. Cancer cachexia in the age of obesity: skeletal muscle depletion is a powerful prognostic factor, independent of body mass index. J Clin Oncol. 2013;31(12):1539-47.
2. Giesinger JM, Kieffer JM, Fayers PM, Groenvold M, Petersen MA, Scott NW, et al. Replication and validation of higher order models demonstrated that a summary score for the EORTC QLQ-C30 is robust. J Clin Epidemiol. 2016;69:79-88.
