# Supplementary material for: Malnutrition risk as a predictor of quality of life and skeletal muscle depletion following upper gastrointestinal cancer diagnosis: A longitudinal analysis
Source: J Nutr Health Aging. 2025 Jul 1;29(9):100623. doi: 10.1016/j.jnha.2025.100623 (PMC12270060; doi:10.1016/j.jnha.2025.100623)
Supplement: Supplementary file 3 [file mmc3.docx]

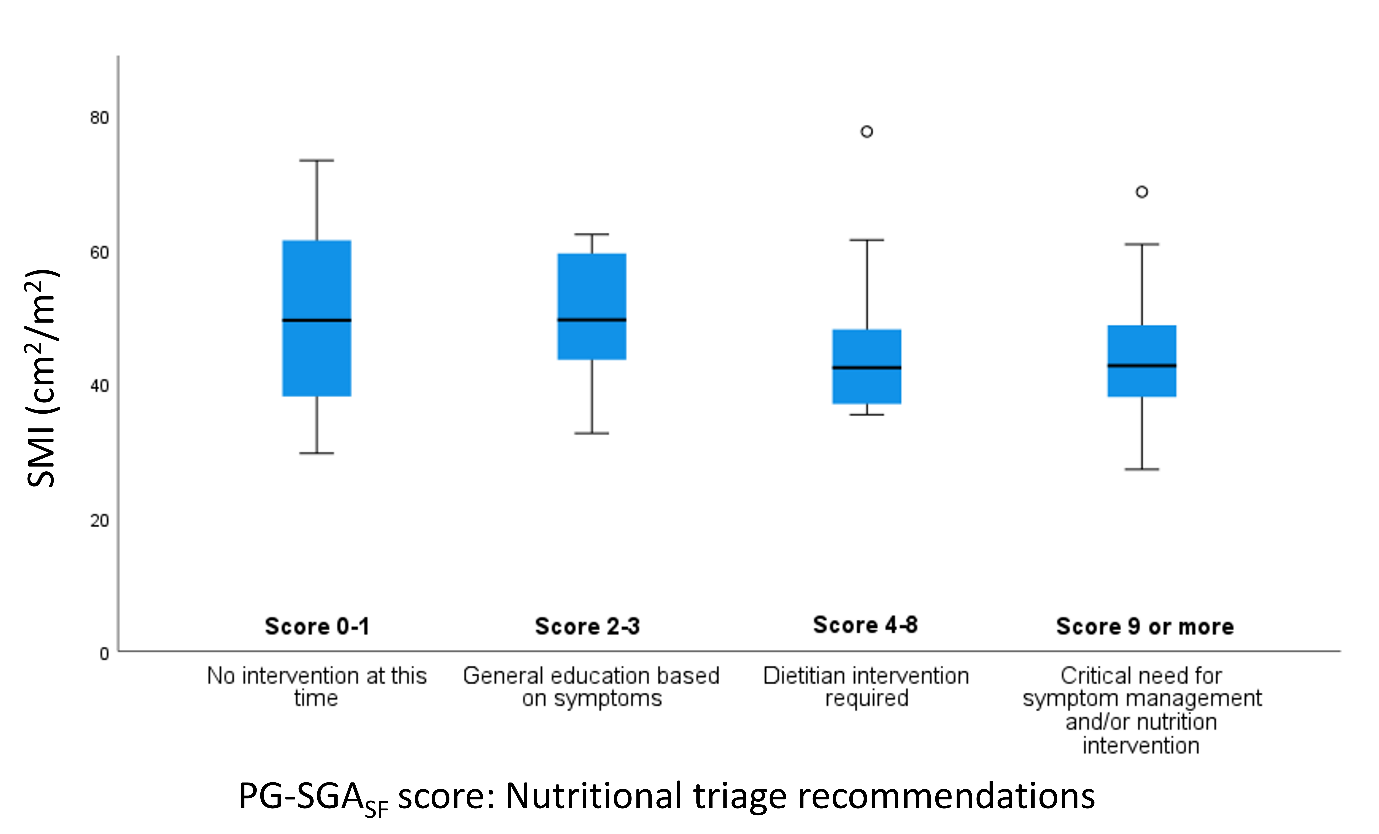


**Supplementary Figure.** Boxplot of median skeletal muscle index (SMI) of participants grouped according to recommended triage categories of the PG-SGA_SF_ tool; n=105; PG-SGA_SF_ Patient Generated Subjective Global Assessment Short Form, higher score indicates worse nutritional condition; J=1488, z=-2.20, p=0.028, r=-0.21
