# Supplementary material for: Malnutrition risk as a predictor of quality of life and skeletal muscle depletion following upper gastrointestinal cancer diagnosis: A longitudinal analysis
Source: J Nutr Health Aging. 2025 Jul 1;29(9):100623. doi: 10.1016/j.jnha.2025.100623 (PMC12270060; doi:10.1016/j.jnha.2025.100623)
Supplement: Supplementary file 4 [file mmc4.docx]

**Association between baseline demographic, phenotypic and malnutrition risk variables with risk of low SMD at diagnosis of upper gastrointestinal cancer**

|  |  | Low SMD  n = 58 | Normal SMD  n = 47 | p | Univariate  OR (95% CI) | p | Multivariate  OR (95% CI) | p |
| --- | --- | --- | --- | --- | --- | --- | --- | --- |
| Age (years)^a^ |  | 70.6 (7.9) | 60.0 (9.0) | <0.001* | 1.16 (1.09-1.23) | <0.001* | 1.16 (1.09-1.24) | <0.001^b^* |
| Sex |  |  |  |  |  |  |  |  |
| Male |  | 35 (60.3) | 36 (76.6) | 0.119 | 1.0 (ref) |  | 1.0 (ref) |  |
| Female |  | 23 (39.7) | 11 (23.4) |  | 2.15 (0.91-5.06) | 0.080 | 2.59 (0.91-7.40) | 0.075^b^ |
| Cancer type |  |  |  |  |  |  |  |  |
| Oesophageal/gastric |  | 38 (65.5) | 28 (59.6) | 0.672 | 1.0 (ref) |  |  |  |
| Pancreatic |  | 20 (34.5) | 19 (40.4) |  | 0.78 (0.35-1.72) | 0.531 |  |  |
| Cancer stage |  |  |  |  |  |  |  |  |
| I-II (resectable/borderline) |  | 25 (23.8) | 27 (25.7) | 0.206 | 1.0 (ref) |  | 1.0 (ref) |  |
| III-IV (unresectable) |  | 33 (31.4) | 20 (19.0) |  | 1.78 (0.82-3.88) | 0.145 | 2.05 (0.75-5.63) | 0.164^b^ |
| BMI (kg/m^2^) |  | 24.4 (22.5-27.1) | 26.4 (22.4-28.6) | 0.292 | 0.99 (0.91-1.07) | 0.759 |  |  |
| Weight loss during 6 months pre-diagnosis (%) |  | 5.6 (0-11.2) | 8.9 (4.0-12.4) | 0.176 | 0.97 (0.91-1.02) | 0.219 | 0.98 (0.91-1.06) | 0.660^b^ |
| PG-SGA_SF_ score |  | 8.5 (2.0-14.25) | 8.0 (3.0-14.0) | 0.867 | 1.01 (0.9-1.073) | 0.757 |  |  |
| < 9 |  | 29 (50.0) | 25 (53.2) | 0.897 | 1.0 (ref) |  |  |  |
| ≥ 9 |  | 29 (50.0) | 22 (46.8) |  | 1.14 (0.53-2.46) | 0.745 |  |  |

n = 105; continuous data reported as median (interquartile range) unless otherwise indicated; BMI body mass index; PG-SGA_SF_ Patient Generated Subjective Global Assessment Short Form; SMD skeletal muscle radiodensity; ^a^mean (standard deviation); ^b^model includes age, female sex, cancer stage, and prior weight loss; *indicates statistically significant result, p<0.05
